# Regenerable Acidity of Graphene Oxide in Promoting Multicomponent Organic Synthesis

**DOI:** 10.1038/s41598-019-51833-2

**Published:** 2019-10-30

**Authors:** Virgilio D. Ebajo, Cybele Riesse L. Santos, Glenn V. Alea, Yuya A. Lin, Chun-Hu Chen

**Affiliations:** 1 Department of Chemistry, National Sun Yat-sen University, Kaohsiung, 80424 Taiwan; 2 Chemistry Department, De La Salle University, Manila, Philippines

**Keywords:** Environmental chemistry, Organic chemistry, Materials for energy and catalysis

## Abstract

The Brønsted acidity of graphene oxide (GO) materials has shown promising activity in organic synthesis. However, roles and functionality of Lewis acid sites remain elusive. Herein, we reported a carbocatalytic approach utilizing both Brønsted and Lewis acid sites in GOs as heterogeneous promoters in a series of multicomponent synthesis of triazoloquinazolinone compounds. The GOs possessing the highest degree of oxidation, also having the highest amounts of Lewis acid sites, enable optimal yields (up to 95%) under mild and non-toxic reaction conditions (85 °C in EtOH). The results of FT-IR spectroscopy, temperature-programed decomposition mass spectrometry, and X-ray photoelectron spectroscopy identified that the apparent Lewis acidity via basal plane epoxide ring opening, on top of the saturated Brønsted acidic carboxylic groups, is responsible for the enhanced carbocatalytic activities involving Knoevenagel condensation pathway. Recycled GO can be effectively regenerated to reach 97% activity of fresh GO, supporting the recognition of GO as pseudocatalyst in organic synthesis.

## Introduction

Carbonaceous materials have emerged as promising green *carbocatalysts* in various organic reactions^1–8^. Graphene oxide (GO), a 2D, carbonaceous material bearing different oxygen functional groups such as alcohol, epoxide, ketones, and carboxylic acid^9–12^, is emerging as a green alternative for organic transformations and particularly superb in acidic carbocatalysis^5,13,14^. The inherent Brønsted acidity of GO, due to the presence of acidic oxygen groups on the edge of GO sheets, has been well exploited in the synthesis of benzylpyrazolyl coumarins^15^, thioacetals^16^, ethers^17^, and other Brønsted acid-catalyzed reactions such as transamidation^18^, Fisher esterification^19–22^, Boc-protection of alcohols^23^, and Kabachnik-Fields reactions^24^. Despite these extensive studies, only the Brønsted acidic nature of GO was recognized. Recently, Szostak^25^ and Bandini groups^26^, demonstrated the success of GO-promoted alkylation of arenes and thiophenes. Among these Friedel-Crafts reactions, the possible activities of Lewis acidity of GO has been suggested. However, studies on the use and understanding of GO capitalizing on its Lewis acidity is very few.

Multicomponent reactions (MCR) involve the combination of three or more compounds in one-pot, which affords rapid formation of small molecules with complex structures. Easy purification, high atom economy, and less waste generation due to a minimal number of synthetic steps make MCR an environmental friendly tool for organic synthesis^27^. Triazoloquinazolinones are attractive target compounds due to their important biological activities as anti-HIV^28^, anticonvulsant^29^, analgesic^30^, and antihypertensive^31^ agents. In the past decade, efforts have been made to synthesize triazoloquinazolinone using different acid catalysts in MCR approach^32–35^, where Lewis acids were proved to be important in facilitating reaction yields. For example, the Lewis acidities of nickel nanoparticles, molecular iodine, and hydrotalcites have been shown to promote the multicomponent synthesis of triazoloquinazolinones effectively^36–38^. However, the drawbacks of non-reusable promoters, toxic solvents, corrosive homogenous acid promoters, and complicated catalysts were involved. Aqueous sustainable, heterogeneous GO materials with the strong acidity and high oxidative degrees are thus a promising candidate to realize high reaction yields, low toxicity solvent, and recyclability.

The reported studies using GO as carbocatalyst in different MCR^39–42^ have shown the loss of active oxygen groups during the reaction, which significantly hinders the reusability. Unclear roles of the involved acidic groups of GO complicate the materials design pathway in improving both the activity and stability in acidity-assisted organic synthesis. In this paper, we investigated the acid role of GO with an activity correlation of a series of multicomponent synthesis of triazoloquinazolinone by systematically varying the amounts of oxygen groups. Extensive spectroscopic results identified that the enhanced activities are the results of additional carbocatalytic contribution due to the Lewis acid sites generated from basal plane epoxide groups, in addition to the contribution from the Brønsted acidic groups at the edges of GO sheets. The Brønsted acid groups showed an upper limit in quantity while the oxidation degrees of GO increase, revealing that the overall reactivity of GO is governed by Lewis acid sites when Brønsted sites reach saturation. We also reported a simple acid regeneration protocol to redeem the acidity of GO and showing the recovery of 97% of its activity compared to fresh ones.

## Results and Discussion

### Characterization of different GO

Due to the recognized importance of oxygen groups on the *carbocatalytic* activity of GO^2^, we hypothesized that higher degrees of oxygenation in GO could lead to a better activity in the multicomponent synthesis of triazoloquinazolinone. We recently reported a method using a preformed acidic oxidizing medium (PAOM) to achieve highly oxidized GO materials with higher quantity of oxygen groups than the conventional Hummers’ method^11^. By pre-mixing concentrated sulfuric acid and permanganate prior to graphite addition, the as-yielded acidic oxidants establish the strong oxidizing environment to introduce higher oxygen groups than the Hummers’ methods. In addition, the diffusion kinetics of PAOM was proven to be much faster in delivering oxidants into the interlayer spacing of graphite crystals. Herein, we followed PAOM method to vary oxidation degrees of GO products using different quantity of KMnO_4_ oxidants, such as 3, 6, and 9 grams, hereby the products were denoted as 1xGO, 2xGO, and 3xGO respectively.

**Figure 1:**
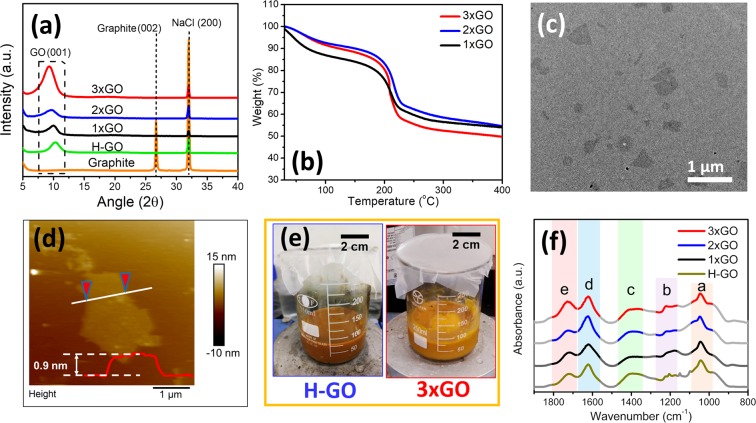
Characterization of prepared GO materials using one-fold, two-fold, and three-fold of KMnO_4_ to vary the oxidation degrees. (**a**) Powder X-ray diffraction patterns with the reference of NaCl peak to indicate the shift of interlayer spacing of GO samples; (**b**) Thermogravimetric analysis results showing the weight loss of GO with varied amounts of oxygen groups; (**c**) TEM images of 3xGO; (**d**) AFM result of 3xGO with monolayer thickness; (**e**) Comparison of physical appearance of H-GO and 3xGO corresponding to different oxidation degrees (**f**) FT-IR spectra of GO materials.

The X-ray diffraction (XRD) patterns of the GO materials using NaCl as internal standard are shown in Fig. 1a. The graphite powder precursor exhibits a characteristic peak at 26.7 (two theta), corresponding to (002) plane of graphite crystal^43^. All of the prepared GO samples show broad XRD peaks in a two theta range of 9–10 degrees, characteristic to GO materials and indicating a successful conversion of graphite to GO^10^. The interlayer distance of (001) peaks of 1xGO, 2xGO, and 3xGO corresponds to 0.881, 0.909, and 0.948 nm, respectively. The Hummers’ method samples (H-GO) show a much smaller (001) d-spacing of 0.851 nm. By increasing the amounts of KMnO_4_ oxidant and the reaction time, the (001) of the PAOM samples shifts to a larger d-spacing due to the higher degrees of oxidation leading to the expansion of interlayer spacing in GO^10,11^. The TGA results show the weight loss (150–280 °C) of 1xGO, 2xGO, and 3xGO to be 29.9%, 34.4%, and 38.8%, respectively (Fig. 1b). 3xGO with the largest weight loss among all GO materials demonstrates the highest quantity of oxygenated functional groups, well agreeing with the XRD data. The TEM images show the 2D characteristic of the as-produced GO samples (Fig. 1c). The AFM results exhibit a typical thickness of 0.9 nm of individual sheets, corresponding to single-layer GO materials (Fig. 1d). As shown in Fig. 1e, the photo comparison of the GO products were acquired right after the H_2_O_2_ addition of the corresponding preparation. The much brighter yellow color in 3xGO suggests the higher oxidation degrees than H-GO, as indicated by the reported literature^11^.

The surface of these GO products is widely decorated by different oxygen functional groups such as hydroxyl groups (-OH), epoxide (C-O-C), and carboxylic acid (-C(O)OH), which can be observed with FT-IR spectroscopy^44^. Fig. 1f shows the FT-IR spectra of the prepared GO materials. The most characteristic FT-IR features are the absorption bands corresponding to C-O-C stretch of epoxide at 1046 cm^−1^ (designated as region a), the C-OH stretching at 1222 cm^−1^ (region b), and the O-H deformation vibration at 1411 cm^−1^ (region c). Besides the ubiquitous O-H stretch which appears as a broad and intense signal at 3300 cm^−1^ (See Fig. S1), another intense peak at 1621 cm^−1^ (designated as region d) can be associated to skeletal vibrations of un-oxidized graphitic domain. The absorption at 1730 cm^−1^ (designated as region e) corresponds to C=O stretching of undissociated carboxylic acid^44–46^. These IR peaks shown in different regions confirm the presence of different oxygen functional groups in the GO samples. Compared to 3xGO, the carbonyl (C=O) stretching peaks (region e) of 1xGO and 2xGO are relatively weaker by referencing the bands in the other regions, further suggesting the greatest oxidation of 3xGO.

To determine the effect of the increasing amount of KMnO_4_ on the oxidation of GO, the elemental analysis data of the GO samples were acquired and summarized in Table 1. The oxygen-to-carbon (O/C) ratios of 1xGO, 2xGO, and 3xGO are 1.367, 1.458, and 1.494, respectively, which are much higher compared to O/C ratio of H-GO (1.257). Furthermore, this clearly shows the increase in oxygenation degrees with higher amount of KMnO_4_. This result can be corroborated with thermogravimetric analysis (TGA). The TGA curves (Fig. 1b) of 1xGO show a significant weight loss of 29.9% at 150–280 °C. This weight loss can be attributed to the thermal decomposition of labile oxygen functional groups such as hydroxyl, epoxide, and carbonyl groups^46–48^. In the same temperature range, both 2xGO and 3xGO exhibit weight loss of 34.4% and 38.8%, respectively. Peak deconvolution of XPS components of GO materials also shows increasing percentage of oxygenated components as the amount of oxidant is increased (see Table S1). All the results above confirm a successful preparation of GO with a decreasing quantity of oxygen functional groups in order of 3xGO > 2xGO > 1xGO > H-GO.

**Table 1:** CHNS elemental analysis of prepared GO Materials.

GO materials	%H	%C	%O	O/C ratio
1xGO	3.154	40.92	55.93	1.367
2xGO	3.062	39.44	57.50	1.458
3xGO	3.183	38.82	58.00	1.494
H-GO	2.978	42.99	54.03	1.257

### Optimization of reaction condition

The activity of the GO materials in promoting multicomponent synthesis were studied for the three-component cyclocondensation reaction between 3-amino-1,2,4-triazole, benzaldehyde, and dimedone. This model multicomponent reaction was studied with respect to different solvents, temperatures, GO loadings, and oxidation degrees of GO materials to achieve the optimal yields (Table 2 and 3). The results show that the product yields are highly dependent on GO loadings as a higher yield was obtained with increasing amount of 1xGO.

**Table 2:** Reaction optimization with various solvents, GO loadings, and temperatures.


^a^Entry	GO Loading [mg]	Solvent	Temp [°C]	Yield [%]^b^
1	25	ACN	85	<5
2	25	EtOH	85	33
3	25	DCM	45	<5
4	25	H_2_O	100	–^b^
5	25	ACN:H_2_O^c^	85	<5
6	25	EtOH:H_2_O^d^	85	<5
7	0	EtOH	85	–^b,e^
8	5	EtOH	85	18
9	50	EtOH	85	37
10	50	EtOH	25	–^b^
11	50	EtOH	45	–^b^
12	50	EtOH	65	35
13	50	EtOH	100	37

^a^Reactions were carried out using 3-amino-1,2,4-triazole **1** (2 mmol), benzaldehyde **2a** (2 mmol), and dimedone **3** (1 mmol) in specific solvents (10 mL) for 4 hours unless otherwise stated; [b] isolated yield, “–” represents no product formed; [c] 2:1 volume ratio; [d] 1:1 volume ratio; [e] after the extended reaction time for 12 hours.

**Table 3:** Activity of different graphene materials.


^a^Entry	Graphene material	Reaction time [mins]	Yield [%]^b^
1	1xGO	240	37
2	2xGO	120	56
3	3xGO	35	95
4	H-GO^c^	240	17
5	r-GO	240	<5
6	Graphite	240	—^b^

^a^Reactions were carried out using 3-amino-1,2,4-triazole **1** (2 mmol), benzaldehyde **2a** (2 mmol), dimedone **3** (1 mmol), 50 mg of graphene materials, and in ethanol solvent (10 mL) at 85 °C; ^b^Isolated yield, “–” represents no product formed; ^c^GO prepared using the Hummers’ method.

Upon testing different solvents for the reaction (Table 2, entry 1–6), only ethanol has shown considerable yields. The ethanol:water mixture results to low yield due to low solubility of the organic reactants. Ethanol could provide both sufficient polarity for homogeneous 1xGO dispersion and higher solubility for the reactants when compared to the other solvents. According to the previous reports, acid promoted synthesis of **4a** was typically carried out in acetonitrile, a more toxic solvent compared to ethanol^36,37^. The amount of 1xGO used in the reaction affects the yields. A higher loading of 50 mg results in a better reaction yield of 37%. Formation of **4a** was not observed in the absence of 1xGO in the synthesis, demonstrating the essential role of GO as the heterogeneous promoter (Table 2, entry 7–9). Carrying out the reaction at temperatures lower than 85 °C resulted in lower yields or no reaction at all. On the other hand, increasing the temperature to 100 °C did not improve the yield either (Table 2, entry 10–13). With the optimal solvent and temperature conditions, the syntheses were further carried out using GO materials with higher oxygenation levels (Table 3). By using 2xGO as the promoter, the yields (56%) are higher than the synthesis using 1xGO with only one-half the reaction time. 3xGO exhibits the highest yields of 95% in just 35 mins (Table 3, entry 3). These results clearly demonstrate the significant influence of GO’s oxidation degrees on the yields of multicomponent synthesis. To further verify this concept, we conducted the reaction with relatively less oxidized GO samples, H-GO, reduced GO (r-GO), and pristine graphite that represents the oxidation-free promoter (see entry 4–6 in Table 3). After the 240 min reactions, the observed yields for H-GO and r-GO were only 17%, and less than 5% of **4a**, respectively, and no formation of **4a** can be observed for the graphite case. NaBH_4_, a weak reducing agent that cannot reduce carboxylic acids, was reported to result in decreased amount of epoxide groups^49^. Comparing 1xGO and its NaBH_4_ reduced product r-GO, a significant lowering of **4a** (Table 3) yields was observed for r-GO which suggests the high reactivity of the basal plane epoxide groups in the reaction. The critical role of oxygen functional groups in facilitating the organic synthesis has been clearly revealed.

### Substrate scope

To evaluate the general applicability of the 3xGO in promoting multicomponent cyclocondensation, we carried out a series of substrate screening (summarized in Table 4) using various aromatic aldehydes (**2b-f**) with 3-amino-1,2,4-triazole (**1**) and dimedone (**3**) to produce the corresponding triazoloquinazolinone derivatives (**4b-f**). Single crystal X-ray of **4c** (Fig. S4) further confirms the structure of the product. The reaction time (e.g., from 35 to 60 mins) required in each synthesis varies with the electronic nature of the aromatic aldehydes (Table 4). All reactions show high yields (78–95%), demonstrating the general applicability of 3xGO in the synthesis of triazoloquinazolinones.

**Table 4:** Multicomponent synthesis of triazoloquinazolinone derivatives.

Entry	Aldehyde	Product	Reaction time (mins)	Yield (%)
1			35	92
2			35	94
3			60	93
4			60	86
5			60	94
6			60	88
7			60	78

### Oxygenated groups on GO sheets

**Figure 2:**
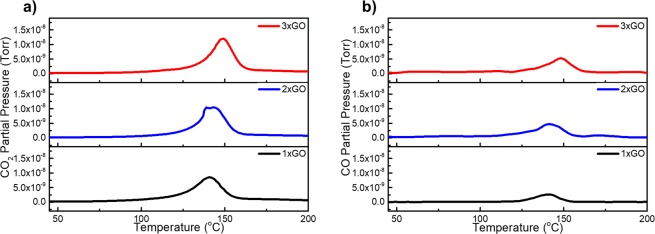
TPD-MS spectra of the gases evolving during the thermal decomposition of the graphene oxide materials with different oxidation degrees: (**a**) CO_2_ (m/z = 44) and (**b**) CO (m/z = 28).

To identify the oxygen species on GO involved in the multicomponent reaction, temperature-programed decomposition mass spectrometry (TPD-MS) measurement was conducted on all the prepared GO materials (Fig. 2). The amount of released species (Torr) is determined by integration of peak area (Table 5). Since GO materials are comprised of hydrogen, carbon, and oxygen atoms only, all the oxygenated groups in GO should be converted to CO and CO_2_ after thermal decomposition. Thus, measurements were particularly focused on CO_2_ and CO evolutions during the TPD-MS experiments as these represent two different oxygenation functional groups: carboxylic and non-carboxylic groups. The amount of these gases during the thermal decomposition of GO may be used to determine the relative population of the oxygen groups.

According to gas cracking pattern data^50^ CO signals can be a cracking fragment of CO_2_ with a relative percentage of 11.4%, this component has been subtracted from the measured intensity of m/z = 28. As shown in Fig. 2, for all the GO materials, CO_2_ evolves at the temperature range of 90 to 170 °C. CO_2_ evolving at this temperature range can be typically attributed to the decomposition of carboxylic acid on the edge of the GO material^51^. Both 2xGO and 3xGO have a similar amount of evolved CO_2_ suggesting the comparable quantity of carboxylic acid groups (Table 5). The saturated quantity of Brønsted carboxylic acid is plausibly due to the limited amounts of accessible carbon at the edges. The CO evolved can be observed at the temperature range of 90 to 160 °C. CO gas detected at this temperature range is due to thermal decomposition of basal plane oxygen groups such as epoxides and alcohols^52,53^. The integrated area for CO evolving from 3xGO (7.76 × 10^−8^ Torr) is greater than that of 2xGO (5.06 × 10^−8^ Torr), indicating the relatively higher quantity of epoxide and alcohol groups on the surface of 3xGO. The same result was obtained from peak deconvolution of XPS spectra for 2xGO and 3xGO which shows an increase in epoxide (C-O-C) component with increasing oxidation (Table S1). The results suggest that the edge groups of carboxylic acid already reached saturation limit of oxidation in 2xGO, which forced the oxidation to occur on the basal plane when the amount of KMnO_4_ was further increased to 9 g (3xGO). The selective increase of epoxide over alcohol groups on basal plane may suggest the occurrence of epoxidation prior to ring-opening that results in alcohol groups. In terms of *carbocatalytic* activity on the synthesis of triazoloquinazolinones, these observations support that the higher activities of 3xGO than 2xGO and 1xGO are due to the greater number of basal plane oxygen groups of epoxide and alcohols on the surface of GO sheets. In addition, the basal plane epoxide and alcohol groups are experimentally supported to be involved in the reaction pathway yielding triazoloquinazolinone compounds based on the observed trend for yield of **4a** using GO materials with different oxidations (Table 3). Peak deconvolution of the XPS spectra (Table S1) shows 3xGO, which gives the highest yield of **4a**, has the highest percentage of epoxide (C-O-C) peak component. The non-saturated characteristics of basal plane groups increasing with the oxidation degrees of GO indicates its dominant role in promoting reaction yields when Brønsted acid sites reaches the upper limits.

**Table 5:** Integrated area of evolved CO_2_ and CO in TPD-MS of GO materials.

GO material	CO_2_ Pressure (Torr)	CO Pressure (Torr)
1xGO	1.57 × 10^−7^	4.32 × 10^−8^
2xGO	1.98 × 10^−7^	5.06 × 10^−8^
3xGO	1.91 × 10^−7^	7.76 × 10^−8^

**Figure 3:**
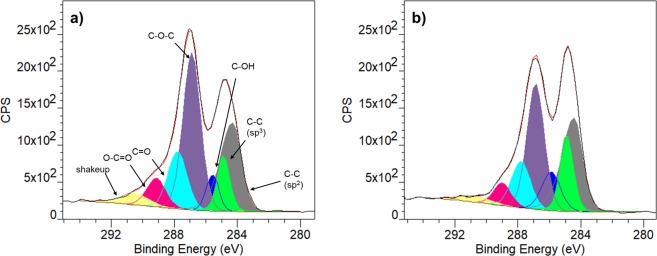
C 1 s XPS spectra of (**a**) 3xGO, and (**b**) 3xGO-5c.

To further confirm the role of oxygen groups in the multicomponent synthesis, we conducted characterization for the comparison between the fresh 3xGO and that after reusing for 5 times (hereby denoted as 3xGO-5c) in the synthesis of **4a**. It is generally accepted that the oxygen functional groups on the surface of GO sheets are the key to hold *carbocatalytic* activities^54^. As shown in Fig. 3, the X-ray photoelectron spectroscopy (XPS) data of the fresh and used 3xGO show the C 1 s signals with peak fitting and deconvolution results. The relative amounts of aromatic carbon (C−C sp^2^, 284.3 eV), aliphatic carbon (C−C sp^3^, 284.9 eV), hydroxyl (C−OH, 285.6 eV), epoxy (C−O−C, 286.9 eV), carbonyl (CO, 287.8 eV), and carboxyl (O−CO, 289.15 eV) were quantified and summarized in Table 6 ^26,55^.

**Table 6:** Abundance percentage of different functional groups on the surface of 3xGO before and after reactions based on Fig. 3.

Sample	O=C-O	C=O	C-O-C	C-OH	C-C (sp^3^)	C-C (sp^2^)
3xGO	7.190	14.48	36.79	6.160	10.00	25.39
3xGO-5c	5.430	12.03	30.25	10.05	14.77	27.46

**Figure 4:**
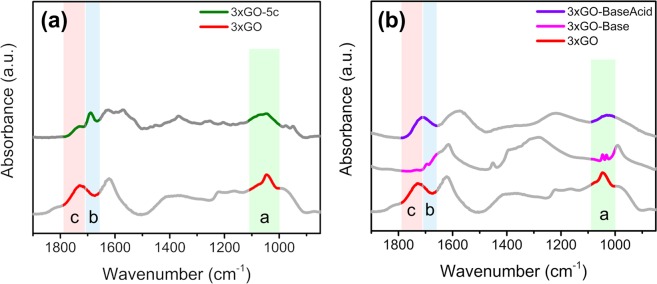
FT-IR spectra of (**a**) fresh 3xGO and recovered 3xGO after five times of use (3xGO-5c), and (**b**) fresh 3xGO, 3xGO-Base, and 3xGO-BaseAcid. The peaks at ~1621 cm^−1^ (graphitic C = C), ~1400 cm^−1^ (-OH vibration), and ~1220 cm^−1^ (C-OH stretch) are not highlighted for clear presentation.

The relative abundance of epoxy groups in 3xGO-5c decreased substantially from 36.8% to 30.3%, while the relative abundance of hydroxyl groups increased from 6.2% to 10.5% suggesting a partial ring opening of epoxide units during the course of the reaction. In addition, the FT-IR spectra of 3xGO and 3xGO-5c (Fig. 4a) shows that, in 3xGO-5c, the weakening (looking at the relative intensity compared to the other peaks) of the stretching band for C=O stretching of undissociated carboxylic acid at 1730 cm^−1^ (region c) accompanied by appearance of new peak at 1690 cm^−1^ (region b) which corresponds to C=O stretching of dissociated carboxylic acid (carboxylate ion)^44^. This suggests the deprotonation of carboxylic acid leading to the formation of carboxylate during the course of the reaction. The C-O-C stretch of epoxide at 1046 cm^−1^ (region a)^46^ also become weaker compared to fresh 3xGO, clearly suggesting the direct participation of both the carboxylic acid and epoxide units in the reaction.

### Mechanism

**Figure 5:**
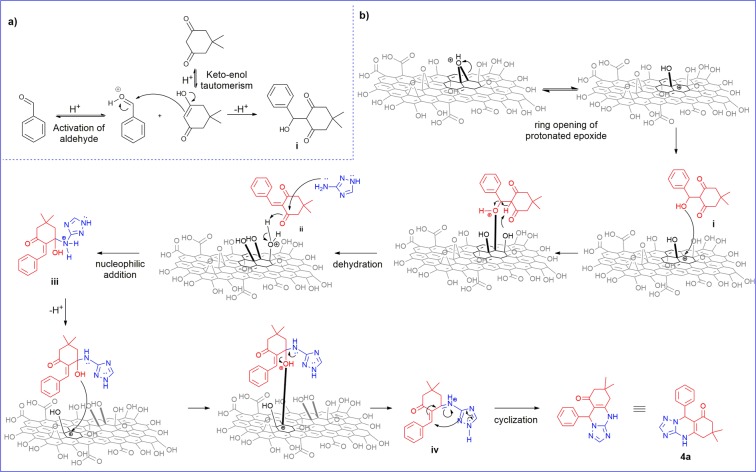
Proposed mechanism for the formation of **4a**. (**a**) 3xGO initiating the reaction by acting as a Brønsted acid. (**b**) role of basal plane alcohol and epoxide of 3xGO. (This figure does not represent the absolute structure of GO. Only the groups involved in the reaction are in bold).

Gathering leads from all the previous results, a mechanism for the formation of **4a** in the presence of carboxylic acid and epoxides on 3xGO is proposed (Fig. 5). It was previously reported in a similar reaction by Dam et. al., that the carboxylic acids of GO are the only functional group involved in the mechanism through: (1) increasing the electrophilicity of the aldehyde and the β-dicarbonyl compound and; (2) promoting the dehydration steps^56^. The XPS and FTIR data, however, suggest that the role of GO is not only limited to the participation of its carboxylic acid group, but also includes the epoxide groups. As shown on Scheme 1a, 3xGO is initially involved as a Brønsted acid initiating the reaction by promoting keto-enol tautomerization of dimedone and activation of benzaldehyde. Reaction of the enol tautomer and the protonated benzaldehyde leads to the formation of intermediate **i**. The role of the epoxides in the mechanism is proposed and summarized in Fig. 5b. Due to the inherent Brønsted acidity of 3xGO, it forms an acidic dispersion which allows the protonation of the epoxide groups at the reaction temperature. Ring opening of the epoxide groups relieves ring strain and forms an alpha-carbocation. The carbocation could act as a Lewis acid aiding dehydration of the beta-hydroxy intermediate **i** to form the Knoevenagel intermediate **ii**^25,26,57,58^. Nucleophilic attack of 3-amino-1,2,4-triazole to **ii** followed by 3xGO promoted dehydration of **iii** and cyclization reaction of **iv** leads to the formation of **4a**. This mechanism not only justifies the overall increase of alcohol functionalities and eventual decrease in epoxide group observed in XPS but also explains the formation of carbonyl peak corresponding to carboxylate ion in FT-IR. In addition, the small increase in the O/C ratio of 3xGO vs 2xGO (Table 1) is mainly associated with the basal plane oxidation increasing the number of Lewis acid sites and enhancing the carbocatalytic activity of 3xGO. This observation also supports the proposed role of GO and the importance of epoxide groups in the mechanism.

**Figure 6:**
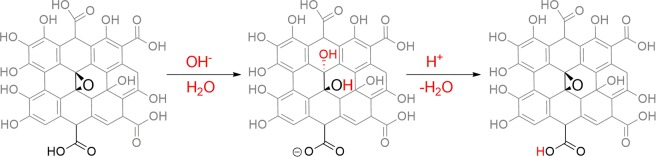
Changes in epoxide and carboxylic acid groups after base and acid treatments on 3xGO (This figure does not represent the absolute structure of GO. Reactions are shown only for the highlighted functional groups for clarity).

To further verify the proposed mechanism where the Brønsted acidic functional groups and the Lewis acidic sites generated from epoxides on 3xGO surface responsible for the activities during the multicomponent reaction, samples of 3xGO treated with a base (denoted as 3xGO-Base) and another subjected to a sequential base-then-acid treatment (3xGO-BaseAcid) were prepared^59^. By subjecting 3xGO to the base treatment, the epoxide groups of the material may undergo ring opening reaction upon nucleophilic attack of OH^−^, leading to the formation of alcohol units as product on the surface of 3xGO-Base, while the subsequent acid treatment may lead to epoxide ring formation via acid catalyzed dehydration (Fig. 6)^60^. The 3xGO was first sonicated in the presence of 1 M NaOH to get 3xGO-Base. Compared to the 3xGO, the FT-IR spectra of 3xGO-Base (Fig. 4b) shows a relatively lowered intensity of C=O of undissociated carboxylic acid at ~1730 cm^−1^ (region c) and the appearance of carboxylate C=O peak at around 1670 cm^−1^ (region b), supporting the deprotonation of carboxylic acid groups. The intense peak corresponding to C-O-C stretch of epoxide which appears at ~1040 cm^−1^ (region a) is absent in the FT-IR spectra of 3xGO-Base. The broad –OH peak at ~3300 cm^−1^ also disappeared after base treatment due to the abstraction of the protons by the strong hydroxide base (Fig. S3). To recover the Brønsted acidic groups and epoxide groups, 3xGO-Base was sonicated in the presence of 1 M HCl to yield 3xGO-BaseAcid. Interestingly, the C=O signal of carboxylic acid at ~1730 cm^−1^ can be regenerated with the weakened signal of carboxylate C=O peaks, revealing a successful reprotonation of the carboxylic acid groups. Broad hydroxyl peak in the FT-IR was also regenerated after acid treatment (Fig. S3). Furthermore, the epoxide C-O-C stretch at ~1040 cm^−1^ was restored signifying the recovery of epoxide units after acid treatment. We carried out the synthesis of **4a** using 3xGO-Base, but it only gave 26% yield of **4a**. By using 3xGO-BaseAcid in the multicomponent reaction, 81% yield of **4a** can be achieved. Current methods for the selective protection of the carboxylic acid groups employs reaction conditions that will lead to ring opening of the epoxide groups which makes isolation of the effect of epoxide groups in the reaction difficult. However, all the obtained results support the importance of both Brønsted acidic groups and generated Lewis acid sites of GO in promoting the multicomponent reaction.

**Figure 7:**
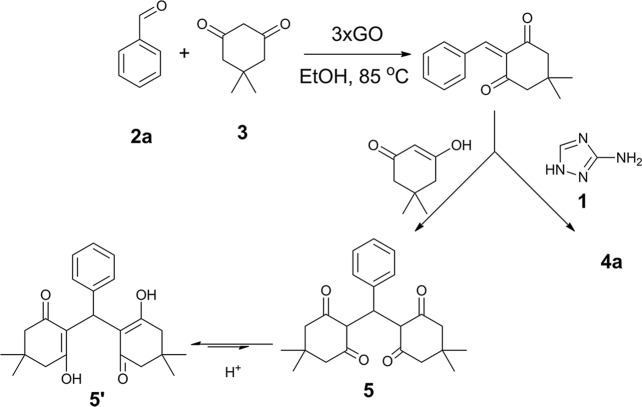
Pathway for the formation of **4a** and side product **5′**.

To date, the mechanism of multicomponent reactions remains questionable^27,61,62^. We were able to isolate the enol **5**′ as the major side product formed during reaction optimization (Table S2) giving an important insight on the mechanism of the reaction. The formation of **5**′ could arise from the Michael-addition of the enol tautomer of dimedone to the Knoevenagel intermediate (Fig. 7) similar to previously reported mechanisms^63,64^. The reaction mechanism for the formation of **5**′ is consistent with our proposed mechanism involving the formation of a Knoevenagel intermediate.

### Reusability study

**Figure 8:**
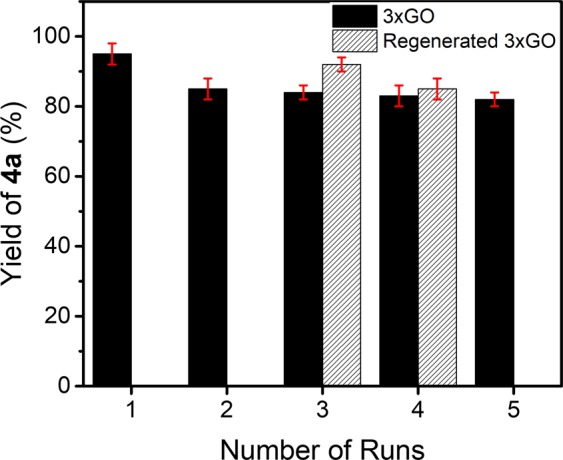
The reusability and regenerability study of 3xGO in multicomponent synthesis of **4a**.

An important advantage of using heterogeneous acid promoters is that they could be conveniently reused without a drastic drop in activity^65^. Reusability studies of 3xGO toward the synthesis of **4a** were conducted and the highest yields for each runs are reported on Fig. 8. 3xGO can be reused for at least 5 times with a slight decrease in the yield (e.g., from 95% for fresh 3xGO to 85% for recycled 3xGO after 5 runs) of **4a**. The consumption of acidic protons and structural changes of 3xGO during the reaction may gradually deactivate 3xGO after each uses suggesting that, contrary to the claims on other *carbocatalytic* systems, 3xGO is not strictly acting as a catalyst in the reaction^66^. The GO samples are more suitable to be considered as a promoter in the organic synthesis. The observed regenerable acidity of 3xGO in the mechanism studies implies that the catalytic activity of used 3xGO may be recovered by acid treatment. A similar acid treatment protocol (sonication with 1 M HCl) was carried out in an attempt to regenerate the used 3xGO. The regenerated 3xGO after 2 runs shows a yield of 92% in the third run, while only 84% yield can be obtained using 3xGO for successive 3 runs of the reaction (Fig. 8). The yield recovery of the acid-regenerated 3xGO is 97% that of the fresh 3xGO. In addition, a slightly higher yield was observed for the acid-regenerated 3xGO (85%) than non-regenerated ones (83%) in the fourth runs. As the structural changes (consumption of acidic protons and ring opening of epoxide groups) on 3xGO excludes it from being a “catalyst” by following conventional definition, the *ex-situ* acid treatment is capable of regenerating the recycled 3xGO to achieve the reaction yields comparable with a fresh one. Together with the acid treatment, 3xGO can nearly recover to its original state ready for the next reaction. In this case, 3xGO cannot be precisely described either as a catalyst or a consumable reagent in organic synthesis. Thus we adopt “pseudocatalyst” to describe GO as a catalyst-like material that require *ex-situ* regeneration to complete its catalytic cycle.

## Conclusion

In this work, both the Lewis-acid sites and Bronsted acidic edges of GO are identified to facilitate triazoloquinazolinone synthesis. Since the quantity of edge carboxylic groups has an upper limit, the increased amount of epoxide Lewis acid sites become the determining factor in achieving highly active GO promoters in organic synthesis, rather than the Bronsted acid sites that tend to be saturated. The 3xGO promoter can be easily regenerated by simple acid treatment, suggesting the high stability of both Lewis and Bronsted acid sites for diverse acid-promoted organic synthesis. The reviewer suggested extending the reaction scope to heterocyclic aldehydes. Preliminary tests using pyridine-3-carbaldehyde yields the corresponding triazoloquinazolinone with the promising yields (see SI). This indicates the wide potential application of 3xGO in heterocyclic aldehyde systems for the multicomponent synthesis of triazoloquinazolinones. Mild reaction conditions, easy work up, and good reusability of 3xGO may fortify the future applications as heterogeneous pseudocatalyst highly active for metal-free, sustainable, less toxic, and green pathway.

## Supplementary information

Regenerable Acidity of Graphene Oxide in Promoting Multicomponent Organic Synthesis
