# Supplementary material for: Regenerable Acidity of Graphene Oxide in Promoting Multicomponent Organic Synthesis
Source: Sci Rep. 2019 Oct 30;9:15579. doi: 10.1038/s41598-019-51833-2 (PMC6821726; doi:10.1038/s41598-019-51833-2)
Supplement: Supplementary file 1 — Regenerable Acidity of Graphene Oxide in Promoting Multicomponent Organic Synthesis [file 41598_2019_51833_MOESM1_ESM.pdf]

Supporting Information
for

Regenerable Acidity of Graphene Oxide in Promoting Multicomponent Organic Synthesis

Virgilio D. Ebajo Jr.[a,b], Cybele Riesse L. Santos [a,b], Glenn V. Alea[b]*, Yuya A. Lin[a], and Chun-Hu Chen[a]*
[a] Department of Chemistry, National Sun Yat-sen University, Kaohsiung, Taiwan 80424
[b] Chemistry Department, De La Salle University, Manila, Philippines
E-mail: glenn.alea@dlsu.edu.ph, chunhu.chen@mail.nsysu.edu.tw

Preparation of Graphene Oxide Materials

Preformed Acidic Oxidizing Medium (PAOM) Method

1xGO was prepared following reported method.[1] In detail, concentrated H_2SO_4 (23 mL, 98%), P_2O_5 (1 g) and $KMnO_4$ (3 g) was mixed in an ice bath. Graphite powder (1 g, Alfa Aesar, 325 mesh) and $NaNO_3$ (0.5 g) was slowly added and the mixture was stirred until homogenous. The reaction vessel was transferred to an oil bath maintained at 35 °C and was stirred vigorously for 1 hour. The reaction vessel was transferred to an ice bath and was allowed to cool. DI water (5x 10 mL) was added carefully. The resulting mixture was heated in an oil bath at 85 °C for 15 min. The reaction was cooled to room temperature and quenched with aqueous H_2O_2 (10 mL, 35%) resulting in a bright yellow mixture. 2xGO was prepared using the same method but using 6 g of $KMnO_4$ and heating for 6 hours at 35 °C instead of 1 hour. 3xGO was prepared using 9 g of $KMnO_4$ and heating for 12 hours at 35 °C.

Hummers' Method

H-GO was prepared using typical procedure as reported by Hummers and Offeman.[2] Graphite powder (1 g, Alfa Aesar, 325 mesh) and $NaNO_3$ (0.5 g) was added to concentrated H_2SO_4 (23 mL) in an ice bath. While the mixture was stirred, $KMnO_4$ (3 g) was slowly added with small portions to prevent a reaction temperature higher than 20 °C. The reaction vessel was transferred to an oil bath maintained at 35 °C and was stirred vigorously for 30 min. The reaction vessel was transferred to an ice bath and was allowed to cool down. DI water (46 mL) was added carefully. The resulting mixture was heated in an oil bath at 98 °C for 15 min. The reaction was cooled to room temperature and was quenched with aqueous H_2O_2 (10 mL, 35%).

Purification of Graphene Oxide Materials
All the GO materials were washed and filtrated with HCl aqueous solution (~4) for five times, followed by a dialysis in water to reach neutral pH conditions under aqueous environment. These GO samples were sonicated for 30 mins and centrifuged at 6000 rpm for 10 min. The resulting aqueous GO dispersion was dried at 60°C overnight and was powdered before use.

Preparation of r-GO
The reduced GO (r-GO) was prepared using similar method reported by Gao et al.[3] The 1xGO (200 mg) was dispersed in DI water (200 mL) first. The pH of the dispersion was adjusted to ~9 to 10 by adding 5 wt% aqueous solution of sodium carbonate. Sodium borohydride (1.6 g) was then added and the mixture was stirred for 2 hours at 80°C. The product was filtered and washed with large amount of DI water. The resultant was oven dried overnight to yield r-GO.

Preparation of 3xGO-Base
An aqueous suspension of 3xGO (35 mL, 15 mg/mL) was mixed with 15 mL of 1.0 M NaOH. The mixture was sonicated for 1 hour, washed with water, ethanol, and acetone. The collected powder were dried overnight at 60°C before use.

Preparation of 3xGO-BaseAcid
An aqueous suspension of 3xGO (35 mL, 15 mg/mL) was mixed with 15 mL of 1.0 M NaOH. The mixture was sonicated for 1 hour, washed with water, ethanol, and acetone. Collected powder were dried overnight at 60°C. The dried powder was sonicated in 1.0 M HCl for 1 hour, washed with water, ethanol, and acetone. Collected powder were dried overnight at 60°C before use.

Regeneration of 3xGO
The recycled 3xGO was sonicated in 1.0 M HCl for 1 hour (10 mL HCl per 50 mg 3xGO), washed with water, ethanol, and acetone. The treated powder was dried overnight at 60°C before use.

Characterization of Graphene Oxide Materials

Transmission electron microscope (TEM) images were obtained by Philips CM200 at 200 kV. X-ray photoelectron spectroscopy (XPS) were prepared by drop casting and drying the diluted ethanol GO suspension onto silicon wafers. The XPS data were recorded with Kratos Axis Ultra DLD with Mg/Al achromatic source. The X-ray diffraction (XRD), elemental analysis (EA), thermogravimetric analysis (TGA), and FTIR samples were powdered samples from oven-dried GO suspension. The XRD patterns were obtained by Bruker D2 Phaser diffractometer with a Cu-Kα X–ray (λ = 1.5418 Å) radiation. EA data were acquired using Vario EL III. TGA results were obtained using TGA Q500. Infrared (IR) spectra were recorded using a Perkin-Elmer Spectrum Two FT-IR Spectrometer with UATR Diamond/ZnSe ATR. Temperature-programmed decomposition–mass spectrometry (TPD-MS) was carried out using Hiden HPR-20 at a heating rate of 10 ^{o}C/min under continuous argon flow.

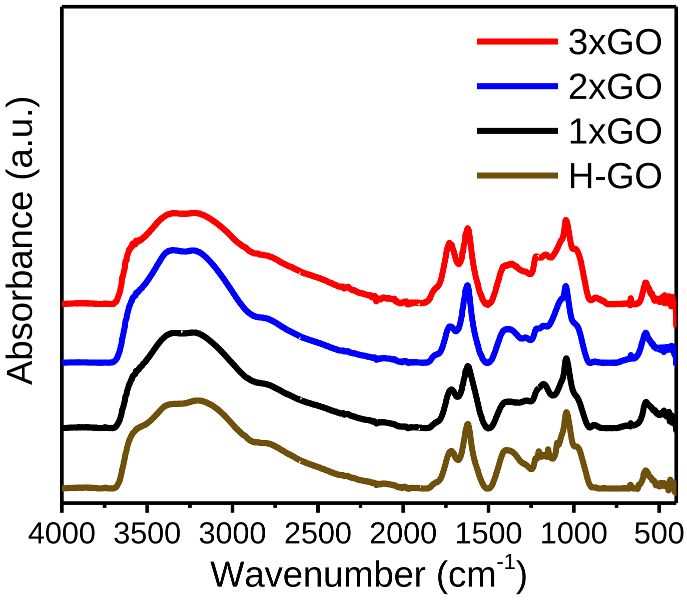

Figure S1. FTIR spectra of the graphene oxide materials.

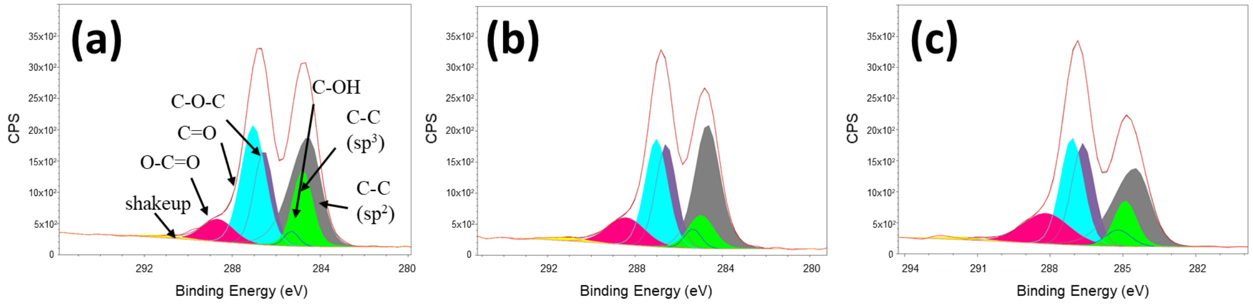

Figure S2. XPS spectra of (a) H-GO, (b) 1xGO, and (c) 2xGO.

Table S1. Abundance percentage of different functional groups on the surface of GO materials based on XPS peak deconvolution.

GO material	O=C-O	C=O	C-O-C	C-OH	C-C (sp^3)	C-C (sp^2)
H-GO[(a)]	6.710	27.08	17.11	2.010	14.83	32.26
1xGO[(a)]	10.04	24.24	21.13	3.260	8.82	32.52
2xGO[(a)]	13.37	23.34	21.37	3.953	10.06	27.90
3xGO[(b)]	7.190	14.48	36.79	6.160	10.00	25.39

[a] Based on Figure S2; [b] Based on Figure 3.

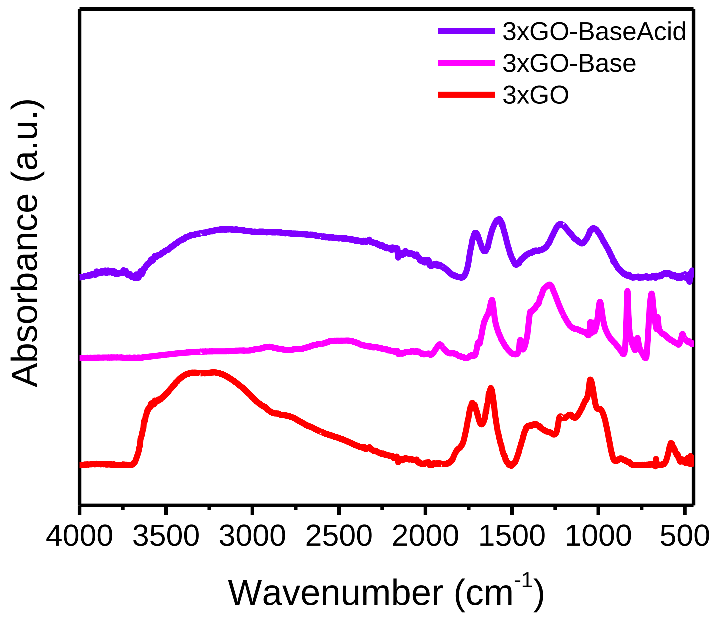

Figure S3. FTIR spectra of 3xGO-Base and 3xGO-BaseAcid.

Table S2. Effect of reactant ratio

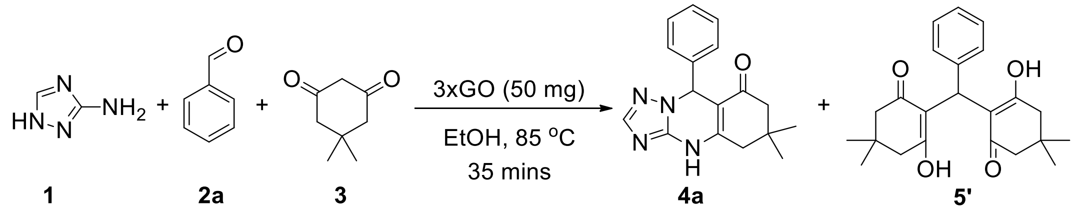

Entry	1 (mmol)	2a (mmol)	3 (mmol)	Yield of 4a (%)	Yield of 5’ (%)
1	1	1	1	0	70
2	2	1	1	0	68
3	1	2	1	0	74
4	1	1	2	0	79
5	2	2	1	95	0
6	2	1	2	0	0
7	1	2	2	78	15

Synthesis of 4a-h

General considerations. All reactions were carried out under air. All commercially available reagents and solvents were used without further purification. Thin layer chromatography (TLC) was carried out using Merck aluminium backed sheets $60F_{254}$ silica gel visualized with a UV lamp (λmax = 254 nm). Proton (^{1}H) and carbon (^{13}C) NMR spectra were recorded using JEOL JNM-ECZ400S (^{1}H: 400 MHz; ^{13}C: 100MHz) or a Bruker Avance 300 MHz-NMR spectrometer (^{1}H: 300 MHz; ^{13}C: 75 MHz), as indicated. Chemical shift values are given in ppm relative to internal DMSO (^{1}H NMR: δ 2.50; ^{13}C NMR: δ 39.51) or $CHCl_3$ (^{1}H NMR: δ 7.24; ^{13}C NMR: δ 77.23). Coupling constants (*J*) were reported in Hz with the following splitting abbreviations: s = singlet, d = doublet, dd = doublet of doublets, t = triplet, ABq = AB quadruplet, and m = multiplet. Mass spectra (MS) were recorded on FT-MS, solariX mass spectrometer (Bruker Daltonics, Germany). Single Crystal Diffraction measurement was done on Xcalibur, Atlas, Gemini ultra diffractometer.

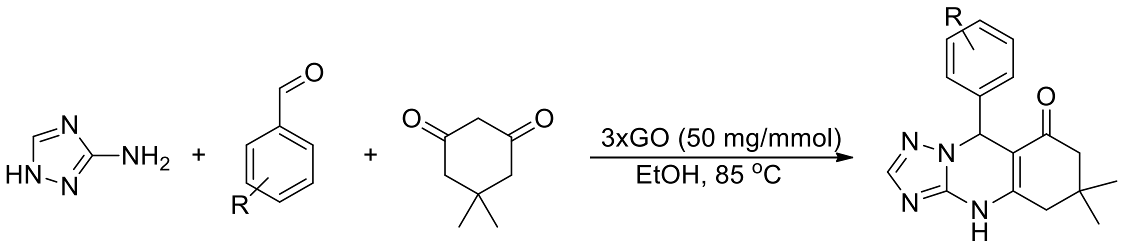

General Procedure. 3-amino-1,2,4-triazole (2 mmol), aldehyde (2 mmol), and anhydrous ethanol (5 mL) were charged to a round-bottomed flask containing a magnetic stir bar. The mixture was

stirred until homogenous. 3xGO (50 mg) was added and the mixture was refluxed at 85 ^{o}C. While refluxing, dimedone (1 mmol) dissolved in 5 mL of anhydrous ethanol was added slowly to the mixture via syringe. The reaction was cooled, centrifuged, and vacuum filtered to separate 3xGO. Organic filtrate was collected and solvent was evaporated. Product was purified by recrystallization in ethanol or isopropyl alcohol. Spectroscopic data of known compounds (**4a-e**, **4g-h**, **5**) are identical to that previously reported. Spectroscpic data (IR, NMR, HR-MS) for the new compound **4f** are provided.

Spectroscopic data of Products:

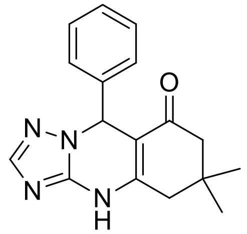

6,6-dimethyl-9-phenyl-5,6,7,9-tetrahydro-[1,2,4]triazolo[5,1-*b*]quinazolin-8(4*H*)-one (4a).[4] White solid. ^{1}H NMR (300 MHz, DMSO-d$_6$) δ 11.11 (s, 1H, NH), 7.68 (s, 1H, H-2), 7.17 – 7.31 (m, 5H, Ar-H), 6.21 (s, 1H, H-9), 2.50 – 2.55 (m, 2H, H-7) ,2.15 (ABq, $\Delta\nu_{AB}$ = 40.3, J_{AB} = 15.0 Hz, 2H, H-5), 1.04 (s, 3H, CH$_3$), 0.96 (s, 3H, CH$_3$); ^{13}C NMR (75 MHz, DMSO-d$_6$) δ 192.9, 150.4, 150.0, 146.9, 141.6, 128.2, 127.7, 126.9, 105.6, 57.9, 49.8, 39.6, 32.2, 28.5, 26.8. MS (ESI) m/z: calculated for $C_{17}H_{19}N_4O^+$ [M+H] 295.16; found 395.15.

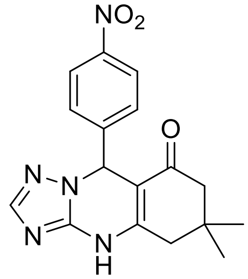

6,6-dimethyl-9-(4-nitrophenyl)-5,6,7,9-tetrahydro-[1,2,4]triazolo[5,1-*b*]quinazolin-8(4*H*)-one (4b).[4] Yellow crystalline solid. ^{1}H NMR (300 MHz, DMSO-d$_6$) δ 11.30 (s, 1H, NH), 8.16 (d, J = 8.6 Hz, 2H, Ar-H), 7.73 (s, 1H, H-2), 7.48 (d, J = 8.6 Hz, 2H, Ar-H), 6.37 (s, 1H, H-9), 2.50 – 2.56 (m, 2H, H-7), 2.15 (ABq, $\Delta\nu_{AB}$ = 40.3, J_{AB} = 15.0 Hz, 2H, H-5), 1.05 (s, 3H, CH$_3$), 0.96 (s, 3H, CH$_3$); ^{13}C NMR (75 MHz, DMSO-d$_6$) δ 193.0, 151.0, 150.4, 148.4, 146.9, 146.9, 128.4, 123.5, 104.7, 57.5, 49.7, 39.5, 32.2, 28.3, 27.0. MS (ESI) m/z: calculated for $C_{17}H_{18}N_5O_3^+$ [M+H] 340.14; found 340.15.

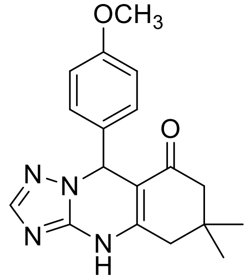

9-(4-methoxyphenyl)-6,6-dimethyl-5,6,7,9-tetrahydro-[1,2,4]triazolo[5,1-*b*]quinazolin-8(4*H*)-one (4c).[4] White crystalline solid. ^{1}H NMR (300 MHz, DMSO-d$_6$) δ 11.06 (s, 1H, NH), 7.66 (s, 1H, H-2), 7.10 (d, J = 8.6 Hz, 2H, Ar-H), 6.83 (d, J = 8.6 Hz, 2H, Ar-H), 6.15 (s, 1H, H-9), 3.70 (s, 3H, OCH$_3$), 2.50 – 2.54 (m, 2H, H-7) , 2.14 (ABq, $\Delta\nu_{AB}$ = 39.2, J_{AB} = 15.0 Hz, 2H, H-5), 1.04 (s, 3H, CH$_3$), 0.97 (s, 3H, CH$_3$); ^{13}C NMR (75 MHz, DMSO-d$_6$) δ 192.9, 158.7, 150.2, 149.9, 146.8, 133.8, 128.1, 113.6, 105.8, 57.3, 55.0, 49.8, 39.5, 32.1, 28.5, 26.8. MS (ESI) m/z: calculated for $C_{18}H_{21}N_4O_2^+$ [M+H] 325.17; found 325.20.

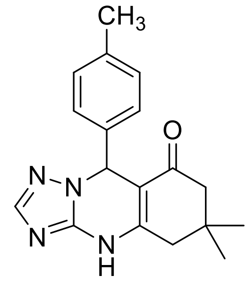

6,6-dimethyl-9-(p-tolyl)-5,6,7,9-tetrahydro-[1,2,4]triazolo[5,1-*b*]quinazolin-8(4*H*)-one (4d).[4] White solid. ^{1}H NMR (300 MHz, DMSO-d_6) δ 11.05 (s, 1H, NH), 7.66 (s, 1H, H-2), 7.07 (s, 4H, Ar-H), 6.15 (s, 1H, H-9), 2.50 – 2.54 (m, 2H, H-7), 2.23 (s, 3H, CH_3), 2.14 (ABq, $\Delta\nu_{AB}$ = 41.4, J_{AB} = 15.0 Hz, 2H, H-5), 1.04 (s, 3H, CH_3), 0.96 (s, 3H, CH_3); ^{13}C NMR (75 MHz, DMSO-d_6) δ 192.9, 150.2, 149.9, 146.8, 138.8, 136.9, 128.7, 126.8, 105.7, 57.6, 49.8, 39.5, 32.1, 28.5, 26.8, 20.6. MS (ESI) m/z: calculated for $C_{18}H_{21}N_4O_2^+$ [M+H] 309.17; found 309.25.

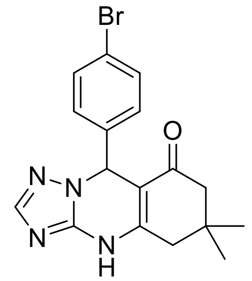

9-(4-bromophenyl)-6,6-dimethyl-5,6,7,9-tetrahydro-[1,2,4]triazolo[5,1-*b*]quinazolin-8(4*H*)-one (4e).[4] White solid. ^{1}H NMR (300 MHz, DMSO-d_6) δ 11.17 (s, 1H, NH), 7.70 (s, 1H, H-2), 7.48 (d, J = 8.3 Hz, 2H, Ar-H), 7.15 (d, J = 8.3 Hz, 2H, Ar-H), 6.20 (s, 1H, H-9), 2.50 – 2.53 (m, 2H, H-7), 2.15 (ABq, $\Delta\nu_{AB}$ = 37.0, J_{AB} = 15.0 Hz, 2H, H-5), 1.04 (s, 3H, CH_3), 0.96 (s, 3H, CH_3); ^{13}C NMR (75 MHz, DMSO-d_6) δ 193.0, 150.6, 150.2, 146.8, 140.9, 131.2, 129.2, 120.8, 105.2, 57.4, 49.8, 39.5, 32.2, 28.3, 26.9. MS (ESI) m/z: calculated for $C_{17}H_{18}BrN_4O^+$ [M+H] 373.07; found 373.15.

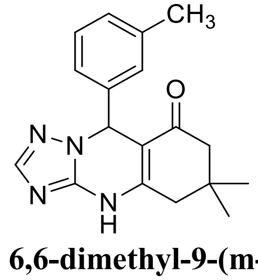

6,6-dimethyl-9-(m-tolyl)-5,6,7,9-tetrahydro-[1,2,4]triazolo[5,1-*b*]quinazolin-8(4*H*)-one (4f). White solid. IR (ν_{max}, solid): 3092, 2920, 1654, 1579, 1543, 1411 1366, 1336. ^{1}H NMR (300 MHz, DMSO-d_6) δ 11.08 (s, 1H, NH), 7.67 (s, 1H, H-2), 7.16 (t, J = 7.7 Hz, 1H, Ar-H), 7.03 (d, J = 7.3 Hz, 2H, Ar-H), 6.95 (d, J = 7.5 Hz, 1H, Ar-H), 6.15 (s, 1H, H-9), 2.50 – 2.55 (m, 2H, H-7), 2.25 (s, 3H), 2.15 (ABq, $\Delta\nu_{AB}$ = 37.9, J_{AB} = 18.0 Hz, 2H, H-5), 1.05 (s, 3H, CH_3), 0.98 (s, 3H, CH_3). ^{13}C NMR (100 MHz, DMSO-d_6) δ 193.0, 150.4, 150.0, 146.8, 141.6, 137.3, 128.4, 128.2, 127.7, 124.0, 105.6, 57.9, 49.8, 32.2, 28.5, 26.8, 21.0. HR-MS (ESI) m/z: calculated for $C_{18}H_{21}N_4O_2^+$

[M+H] 309.17099; found 309.17086.

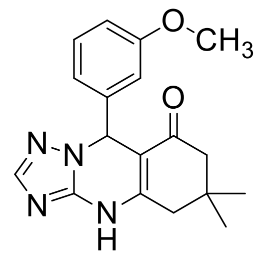

9-(3-methoxyphenyl)-6,6-dimethyl-5,6,7,9-tetrahydro-[1,2,4]triazolo[5,1-*b*]quinazolin-8(4*H*)-one (4g).[5] White crystalline solid. ^{1}H NMR (400 MHz, DMSO-d$_6$) δ 11.13 (s, 1H, NH), 7.69 (s, 1H, H-2), 7.19 (t, J = 8.1 Hz, 1H, Ar-H), 6.79 – 6.81 (m, 1H, Ar-H), 6.72 – 6,74 (m, 2H, Ar-H), 6.18 (s, 1H, H-9), 3.70 (s, 3H, OCH_3), 2.50 – 2.60 (m, 2H, H-7), 2.15 (ABq, $\Delta\nu_{AB}$ = 53.7, J_{AB} = 16.0 Hz, 2H, H-5), 1.04 (s, 3H, CH_3), 0.98 (s, 3H, CH_3). ^{13}C NMR (100 MHz, DMSO-d$_6$) δ 193.0, 159.1, 150.5, 150.1, 146.9, 143.1, 129.4, 119.1, 113.2, 112.7, 105.5, 57.8, 55.0, 49.8, 32.2, 28.6, 26.8. MS (ESI) m/z: calculated for $C_{18}H_{21}N_4O_2^+$ [M+H] 325.17; found 325.20.

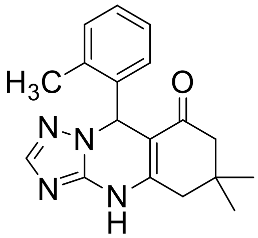

6,6-dimethyl-9-(o-tolyl)-5,6,7,9-tetrahydro-[1,2,4]triazolo[5,1-*b*]quinazolin-8(4*H*)-one (4h).[6] White solid. ^{1}H NMR (400 MHz, DMSO-d_6) δ 11.09 (s, 1H, NH), 7.63 (s, 1H, H-2), 7.11 – 7.13 (m, 1H, Ar-H), 7.07 – 7.09 (m, 2H, Ar-H), 6.97 – 6.99 (m, 1H, Ar-H), 6.44 (s, 1H, H-9), 2.55 – 2.57 (m, 5H, CH_2, CH_3), 2.14 (ABq, $\Delta\nu_{AB}$ = 57.8, J_{AB} = 16.0 Hz, 2H, H-5), 1.05 (s, 3H, CH_3), 1.00 (s, 3H, CH_3). ^{13}C NMR (100 MHz, DMSO-d_6) δ 193.1, 150.5, 150.0, 146.6, 140.4, 135.6, 130.1, 127.5, 126.7, 126.3, 106.2, 54.2, 49.8, 32.3, 28.5, 26.9, 19.0. MS (ESI) m/z: calculated for $C_{18}H_{21}N_4O^+$ [M+H] 309.17; found 309.20.

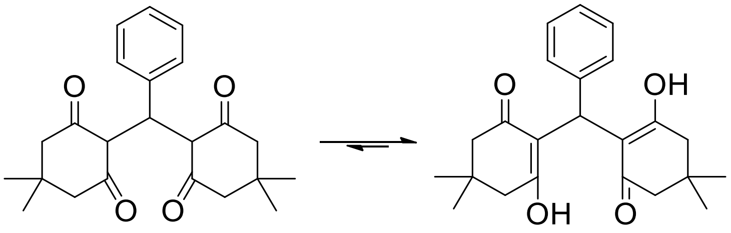

2,2'-(phenylmethylene)bis(5,5-dimethylcyclohexane-1,3-dione) (5).[7] White crystalline solid. ^{1}H NMR (300 MHz, $CDCl_3$) δ 11.82 (br s, 1H, OH), 7.19 (dd, *J* = 8.2, 6.5 Hz, 2H, H-3/ H-5), 7.10 (t, *J* = 6.8 Hz, 1H, H-4), 7.02 (d, *J* = 8.0 Hz, 2H, H-2/H-6), 5.47 (s, 1H, H-7), 2.21 – 2.42 (m, 8H, H-4/H-4'/H- 6/H-6), 1.16 (s, 6H, CH_3), 1.03 (s, 6H, CH_3). ^{13}C NMR (75 MHz, $CDCl_3$) δ 138.3, 128.4, 127.0, 126.0, 115.8, 77.2, 46.7, 33.0, 31.6, 29.8, 27.6. MS (ESI) m/z: calculated for $C_{23}H_{29}O_4^+$ [M+H] 369.21; found 369.20.

NMR spectra of compounds:

^{1}H NMR (300 MHz, DMSO-d$_6$)

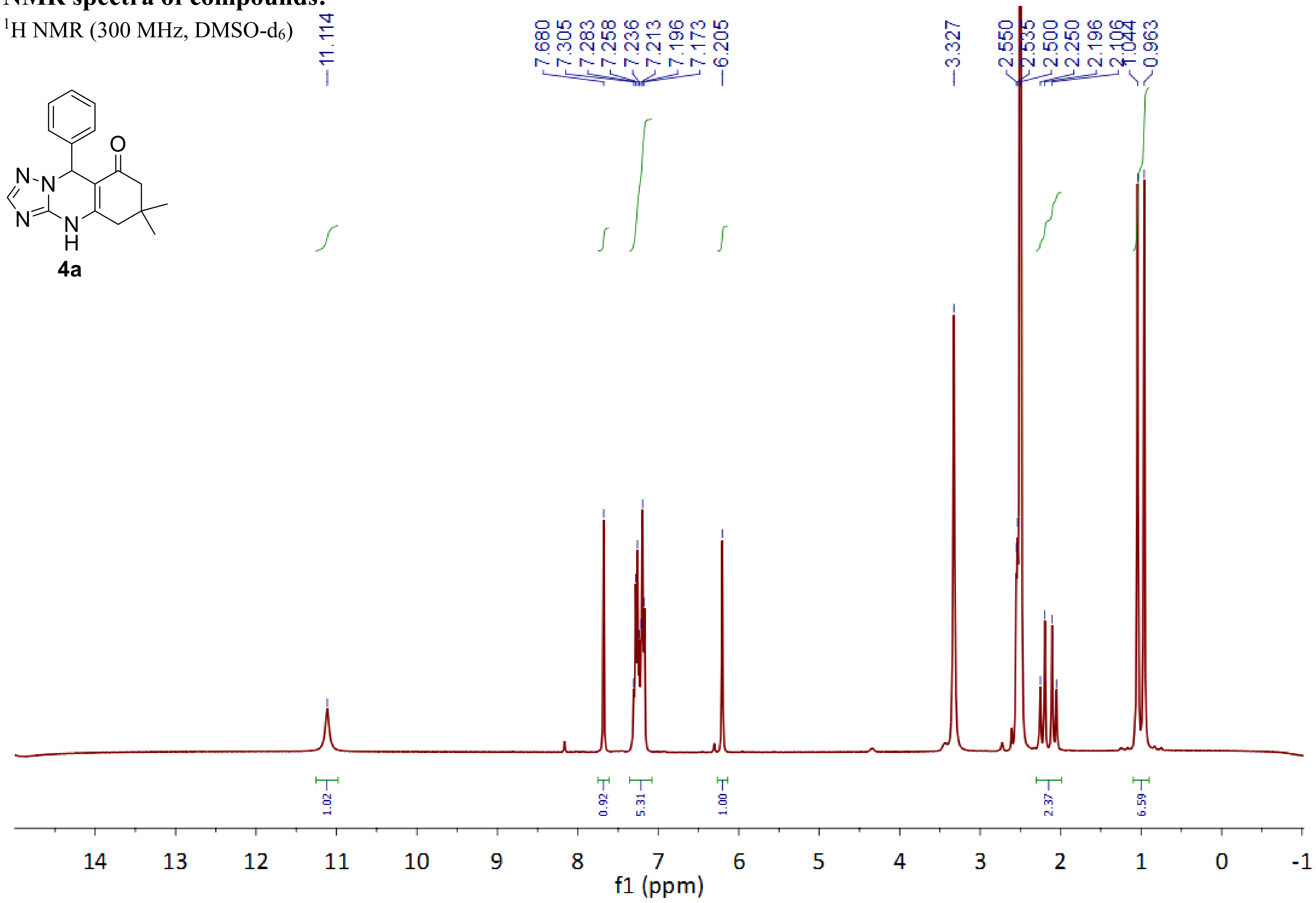

^{13}C NMR (75 MHz, DMSO-d_6)

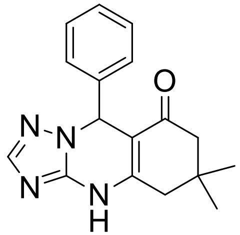

4a

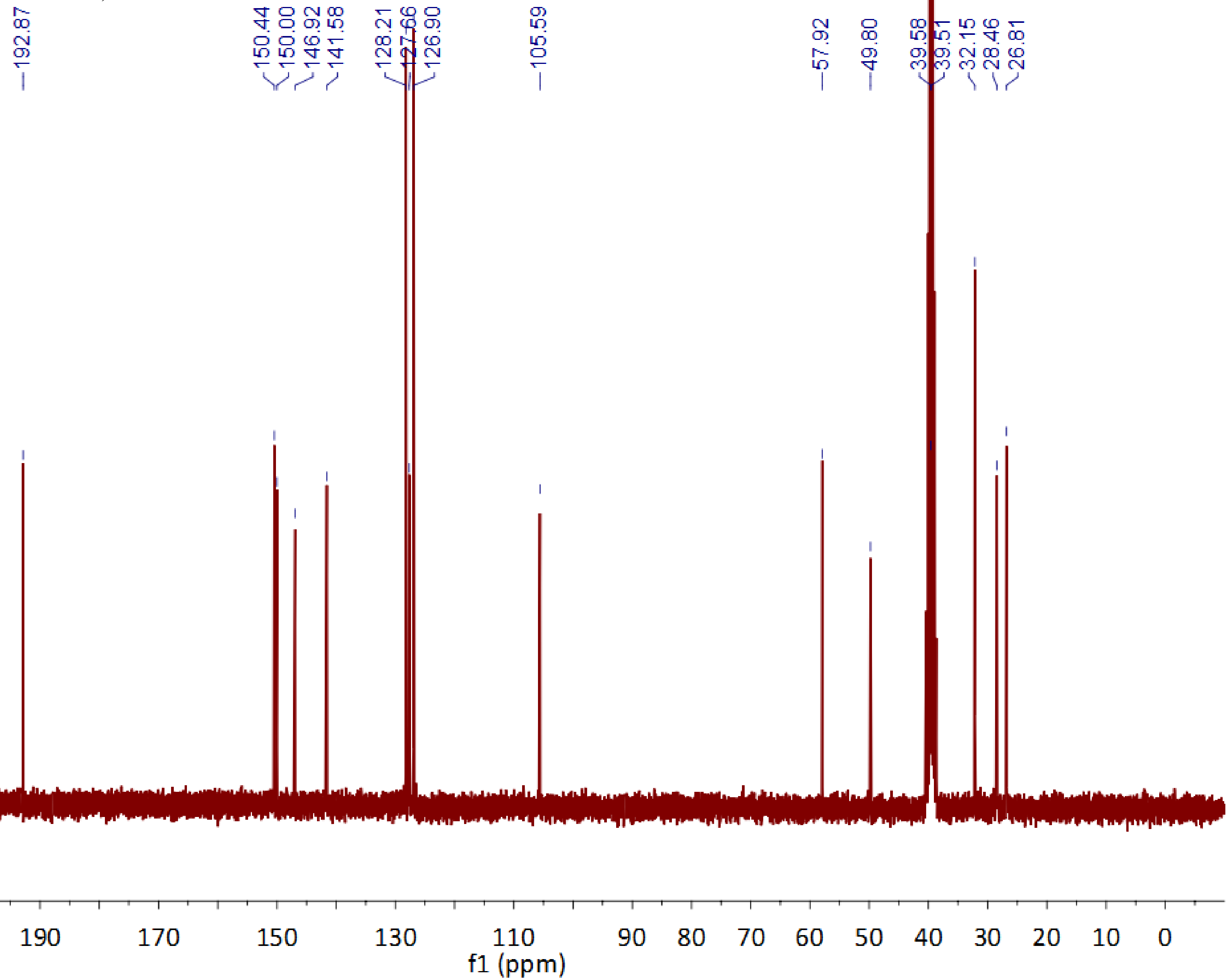

^{1}H NMR (300 MHz, DMSO-d_6)

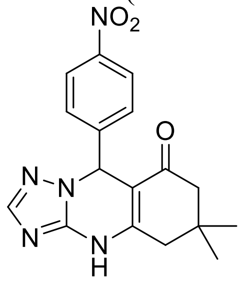

4b

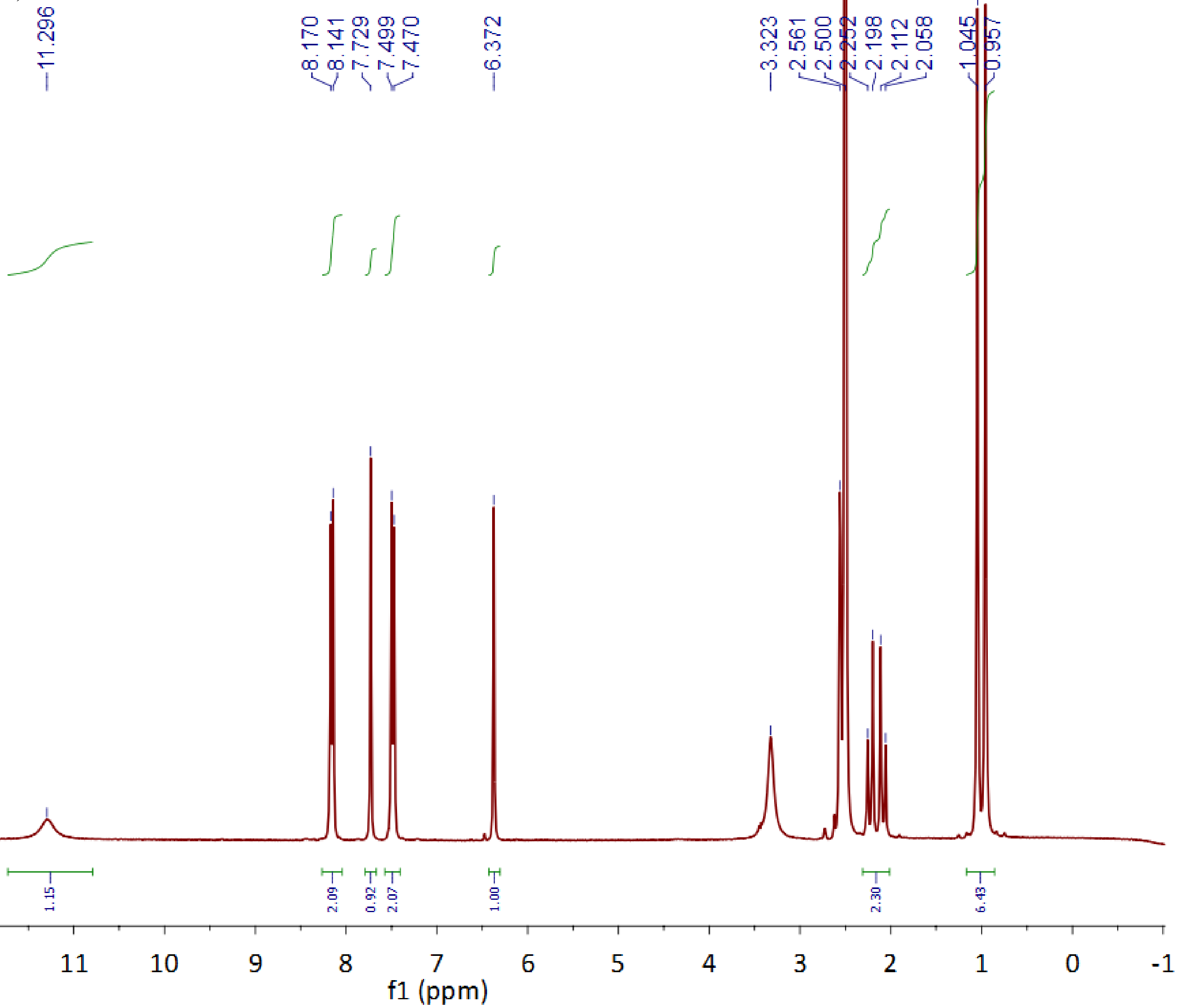

^{13}C NMR (75 MHz, DMSO-d_6)

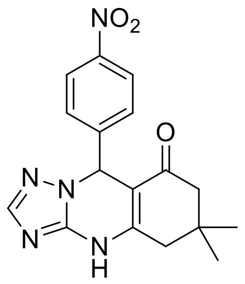

4b

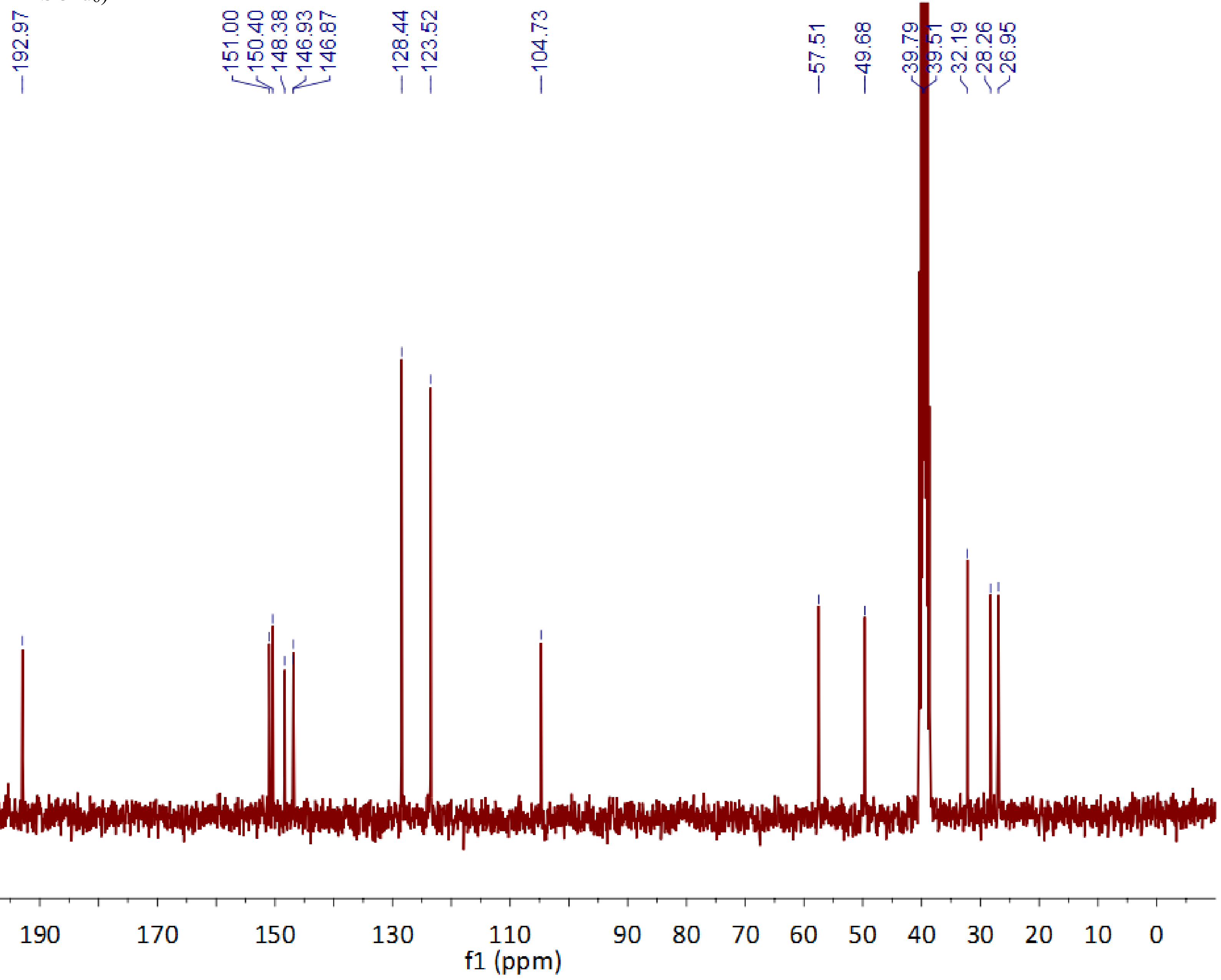

^{1}H NMR (300 MHz, DMSO-d_6)

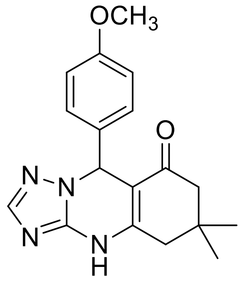

4c

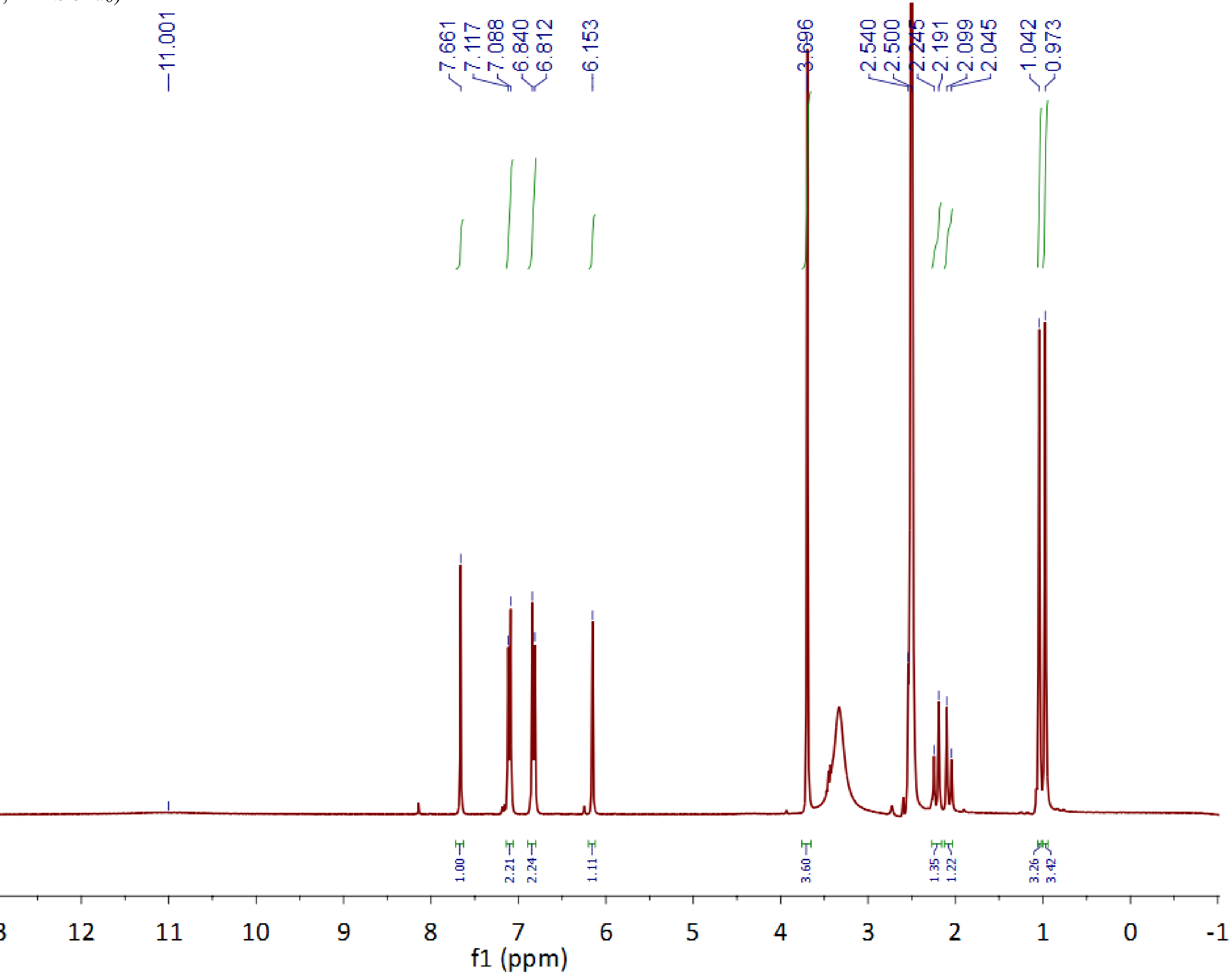

^{13}C NMR (75 MHz, DMSO-d_6)

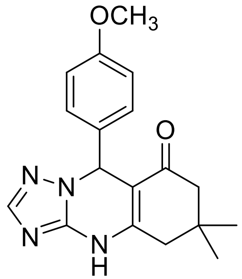

4c

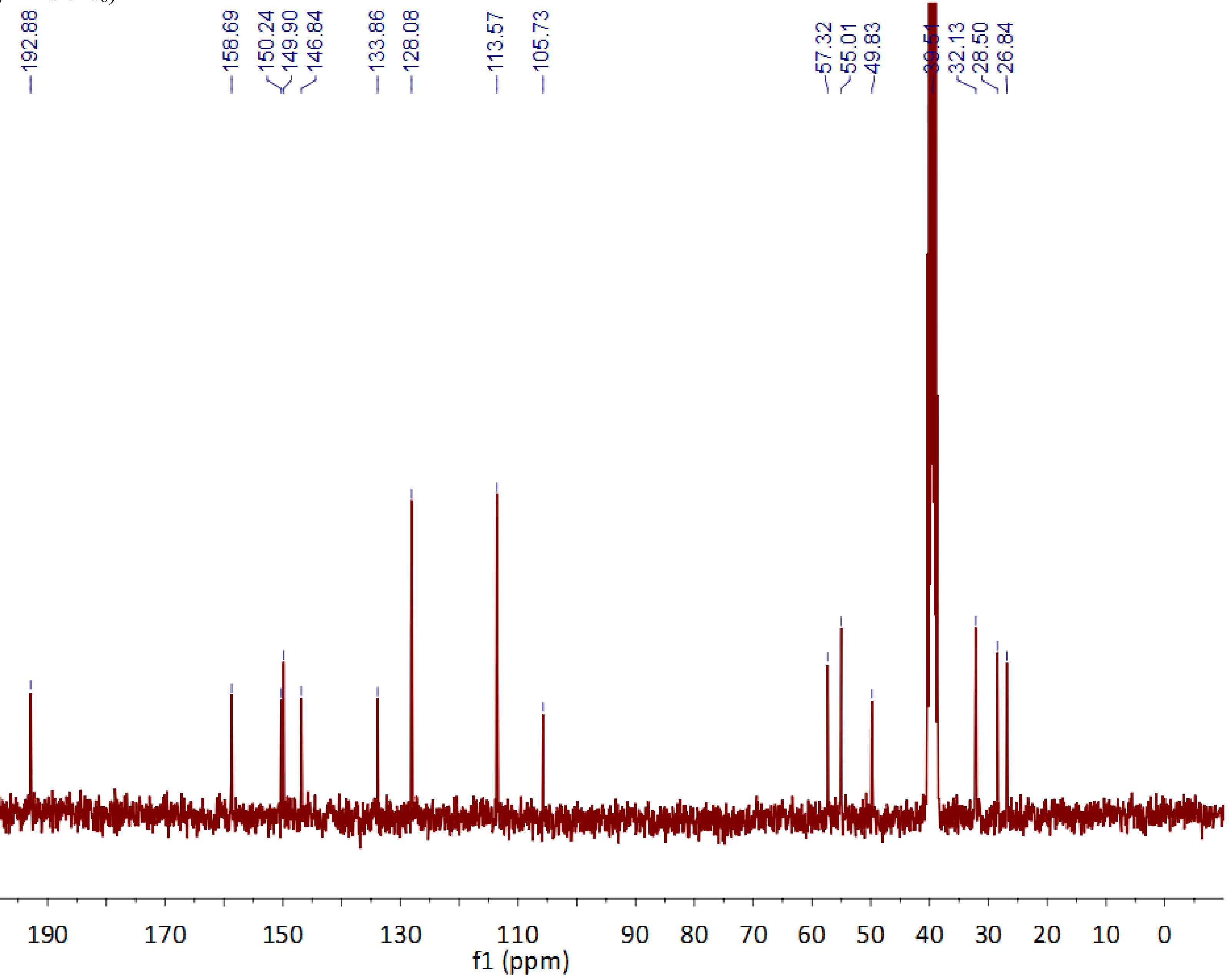

^{1}H NMR (300 MHz, DMSO-d$_6$)

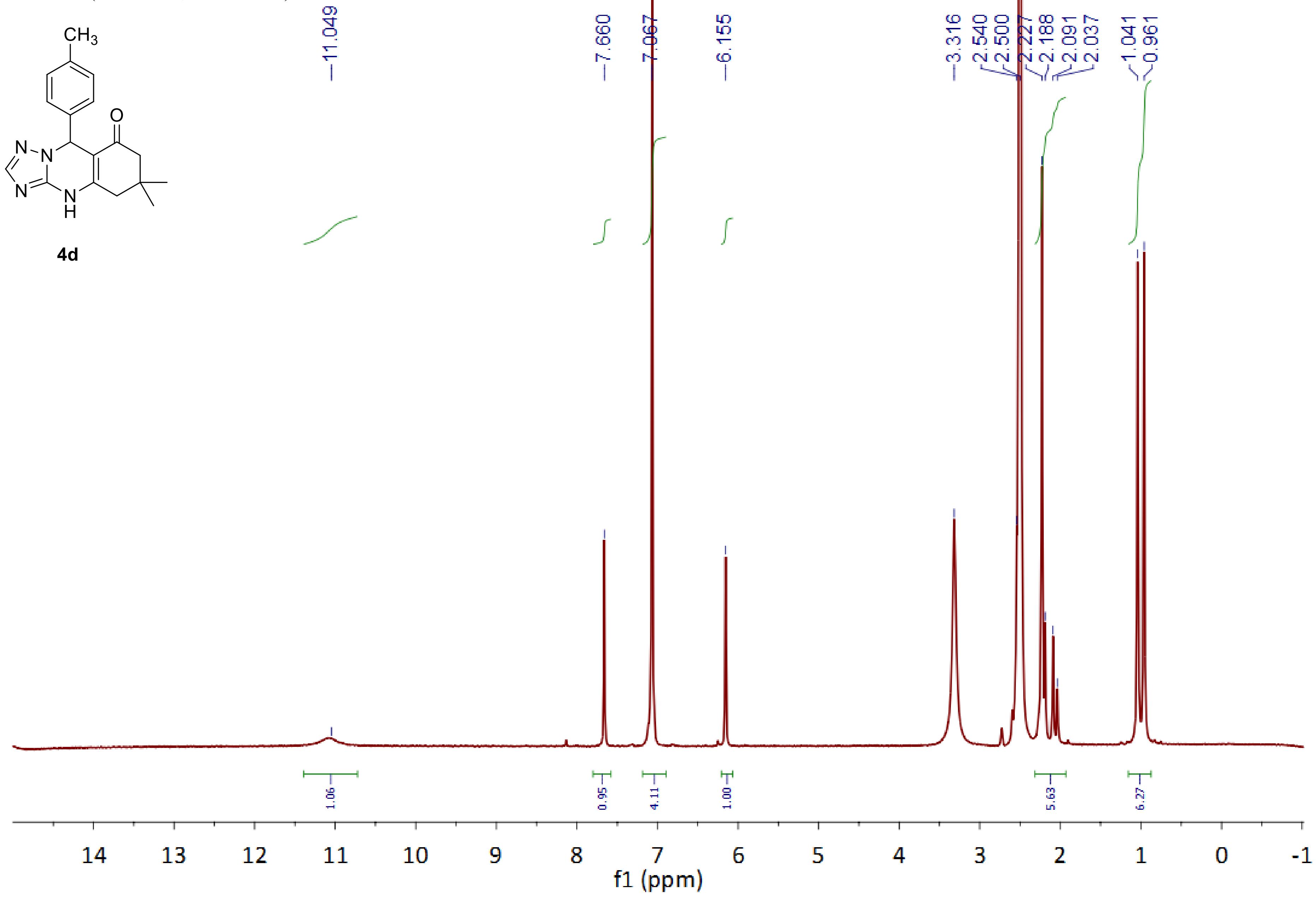

^{13}C NMR (75 MHz, DMSO-d_6)

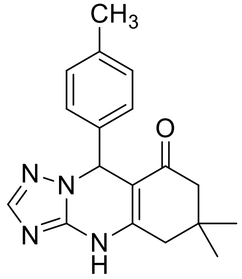

4d

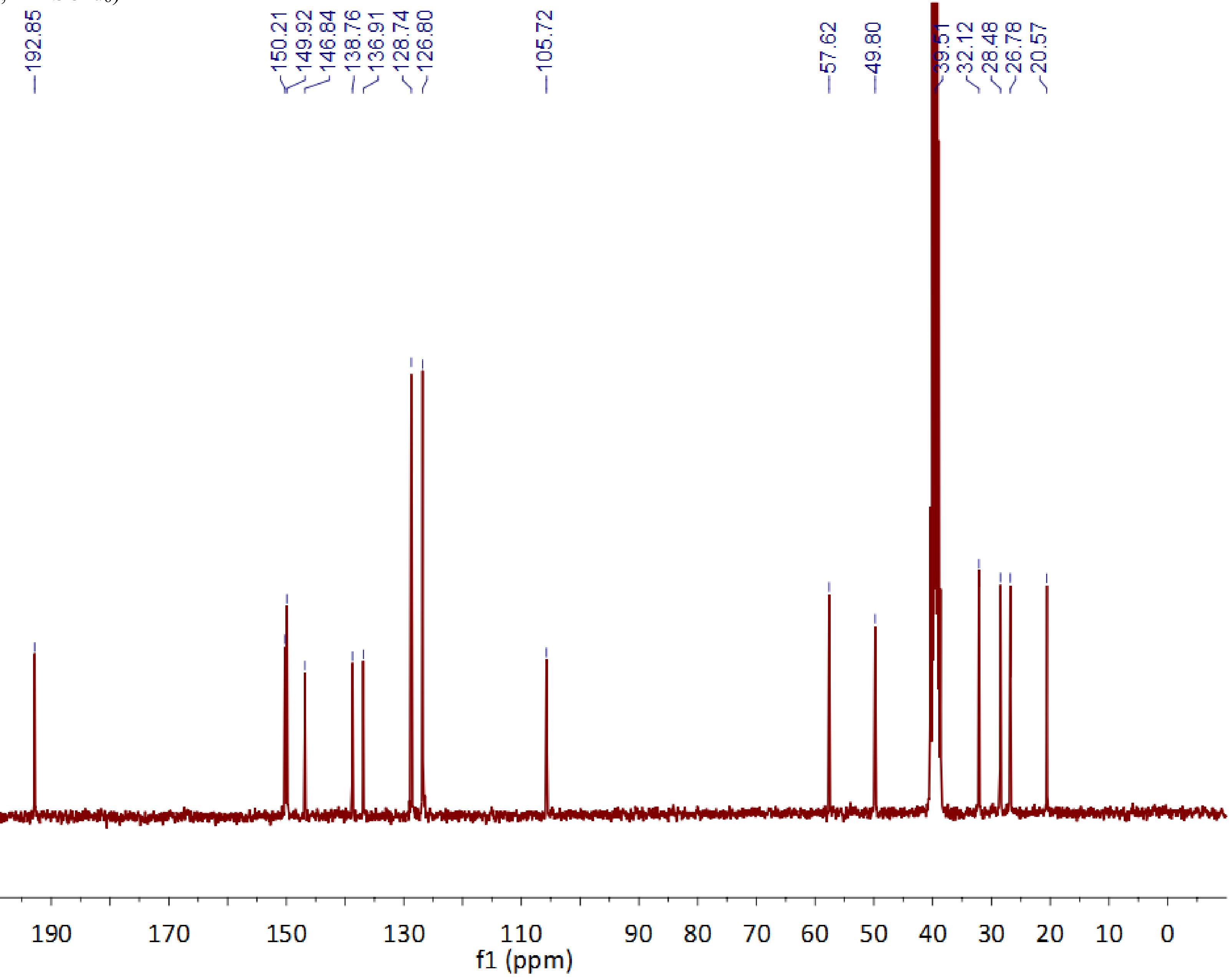

¹H NMR (300 MHz, DMSO-d_6)

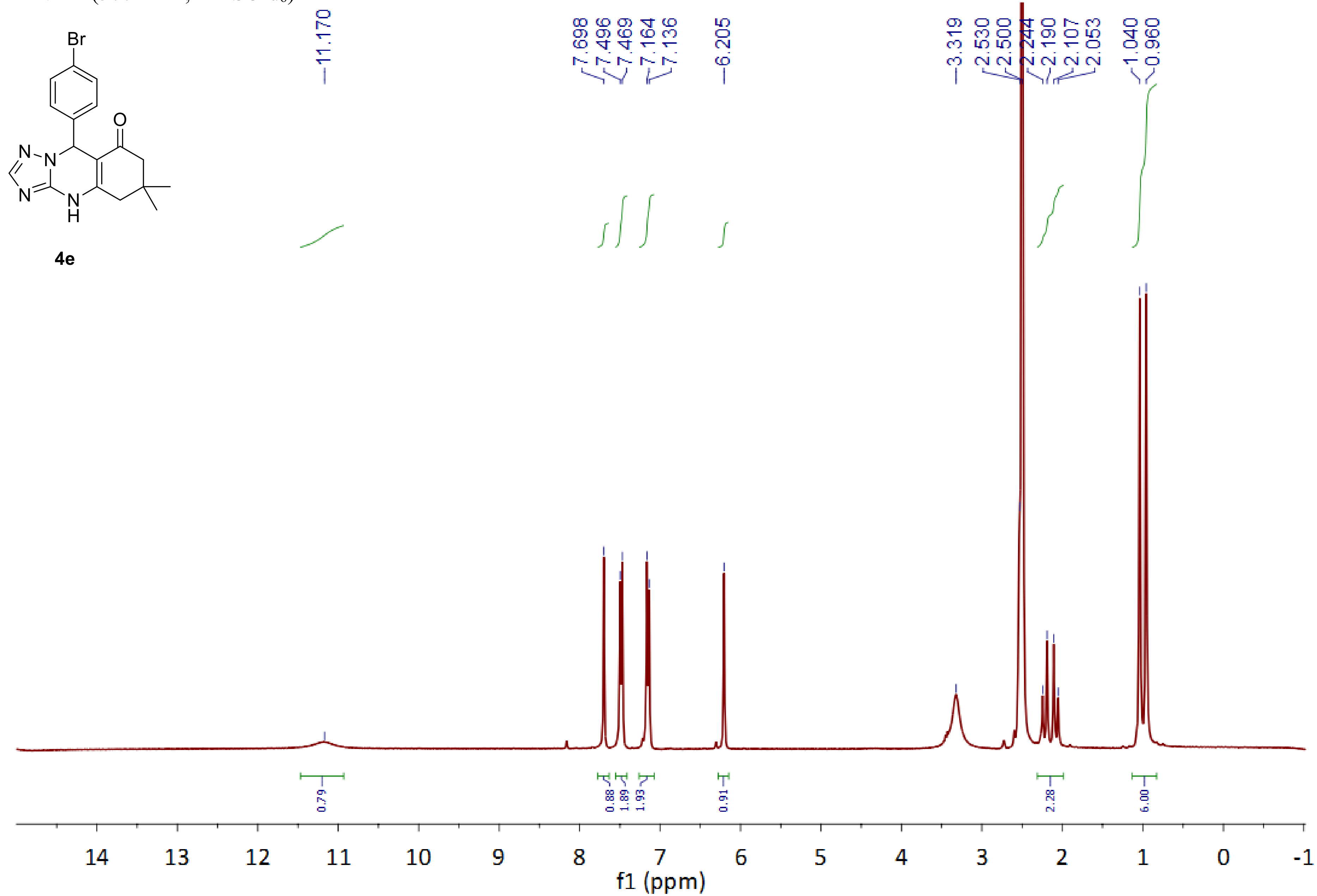

^{13}C NMR (75 MHz, DMSO-d6)

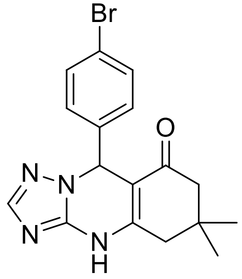

4e

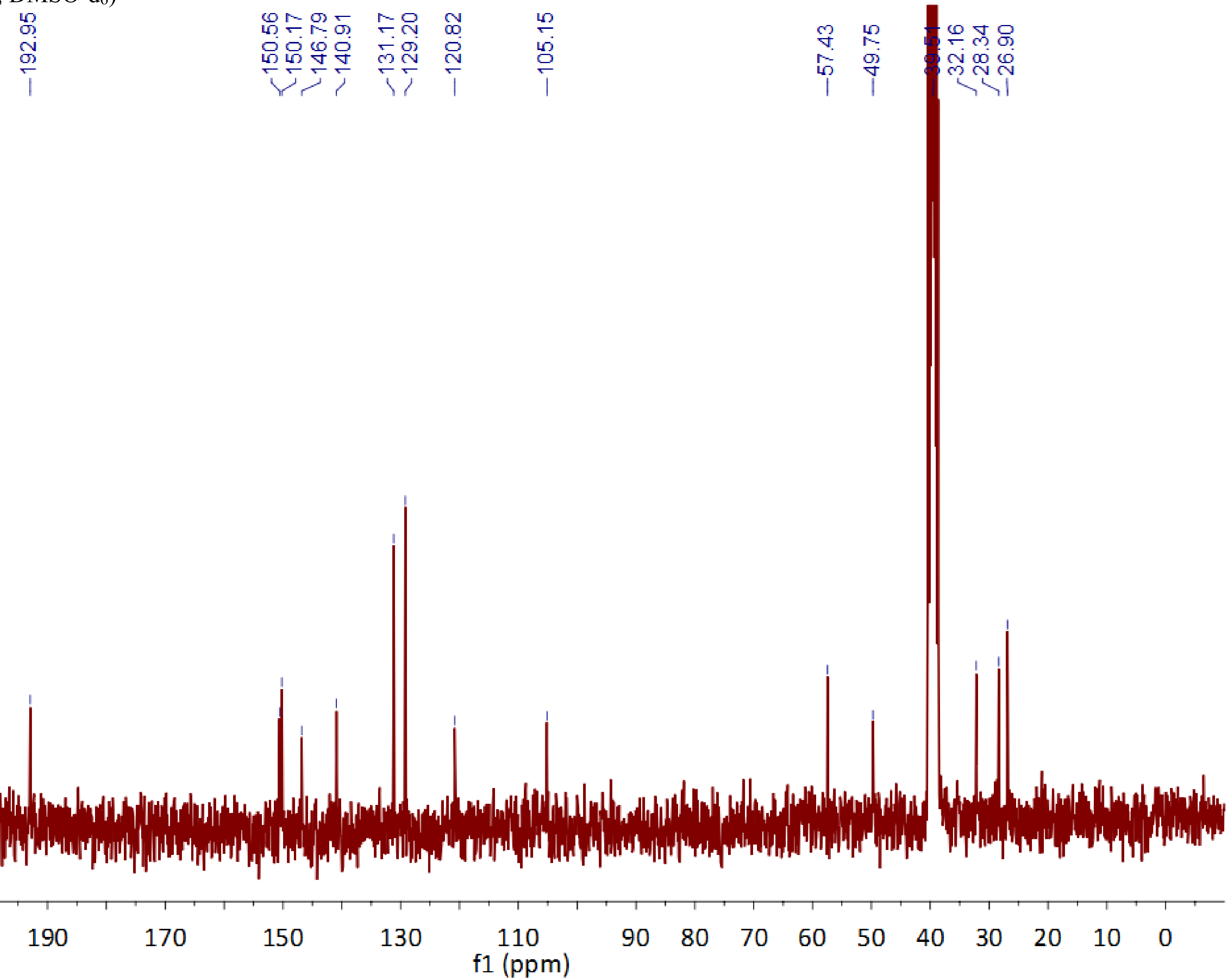

^1H NMR (300 MHz, DMSO-d_6)

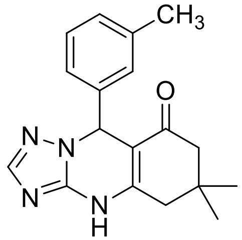

4f

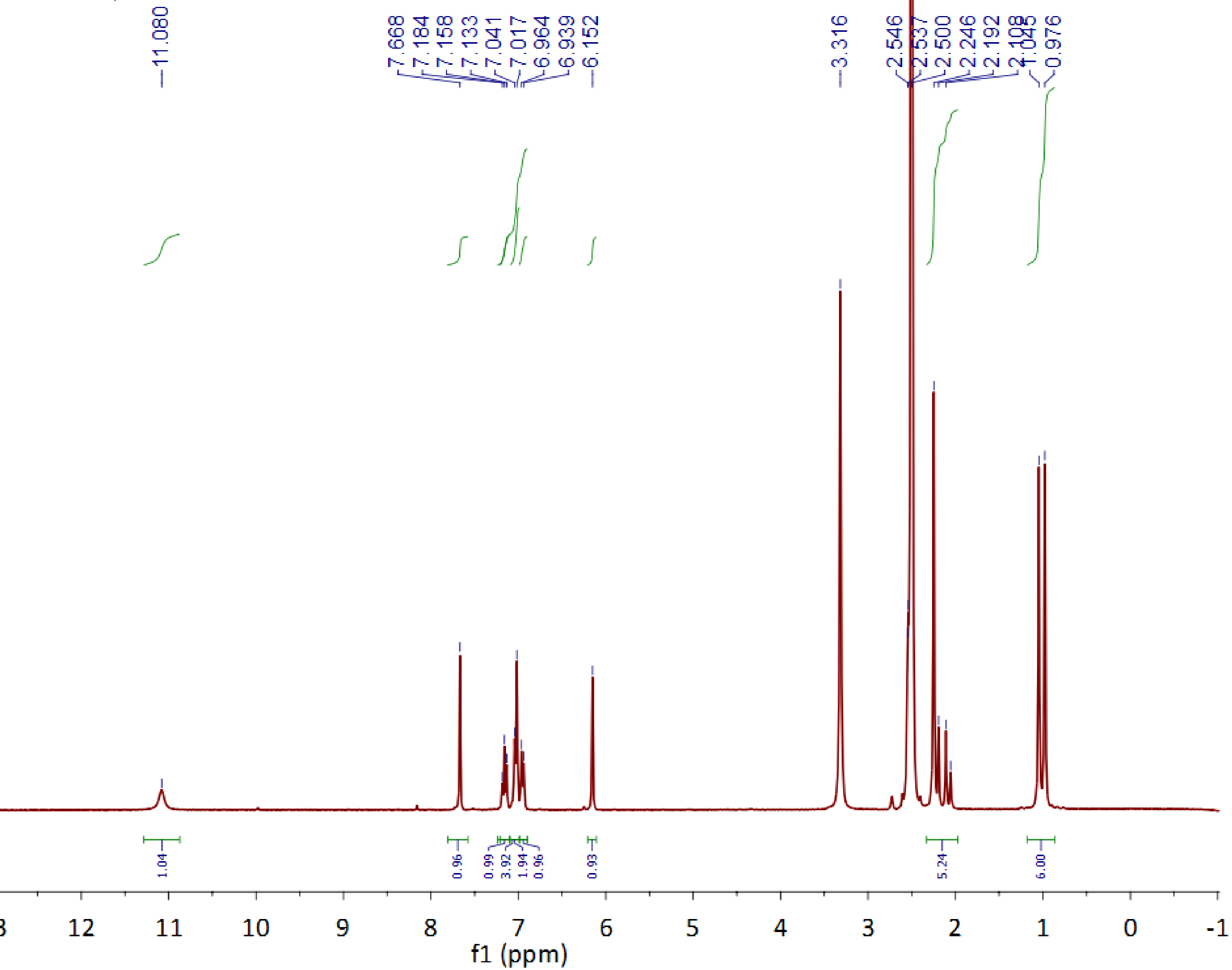

^{13}C NMR (100 MHz, DMSO-d_6)

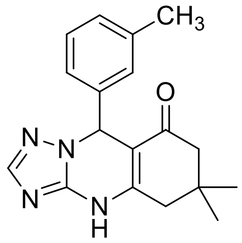

4f

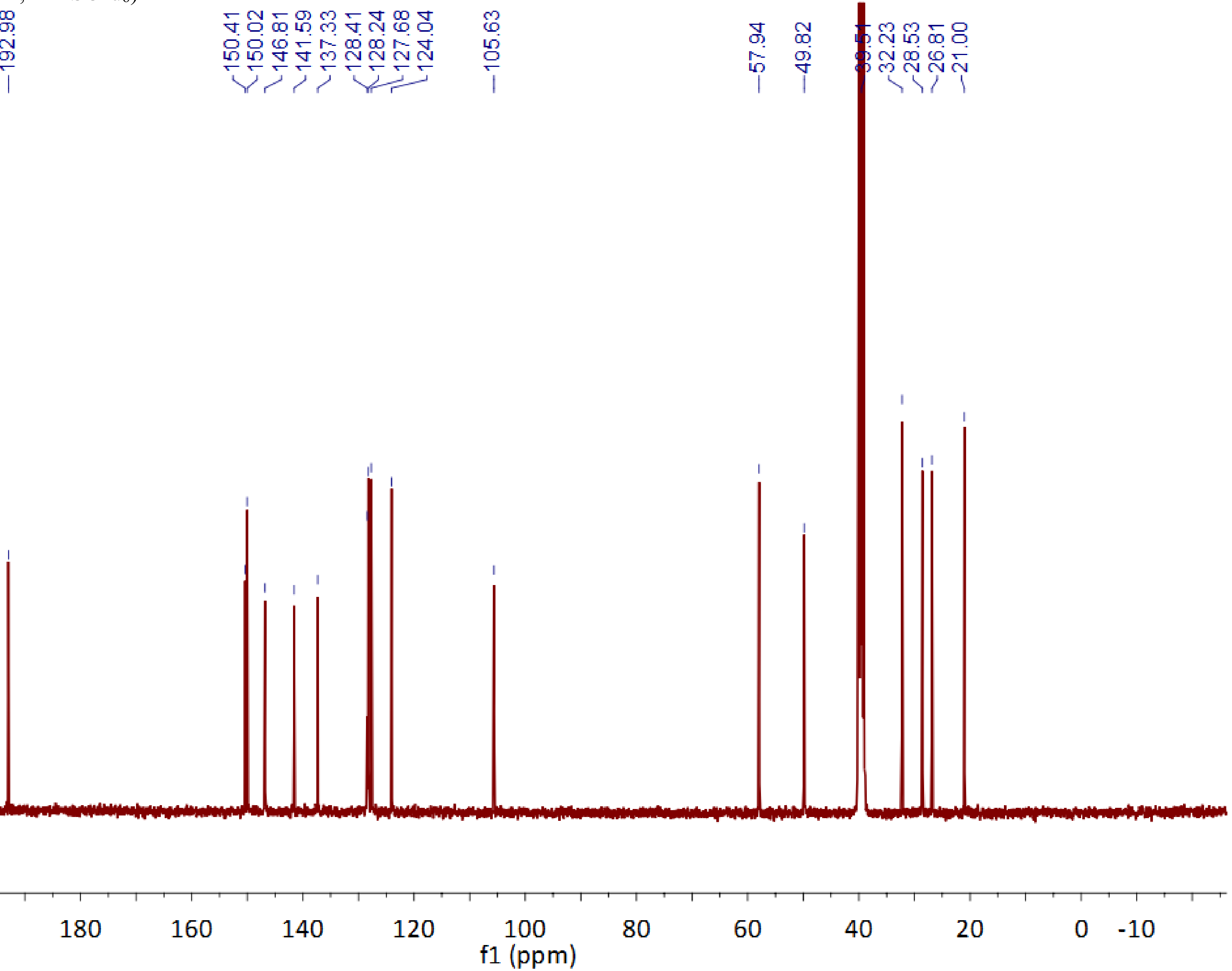

^{1}H NMR (400 MHz, DMSO-d_6)

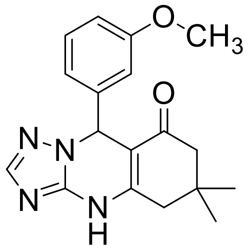

4g

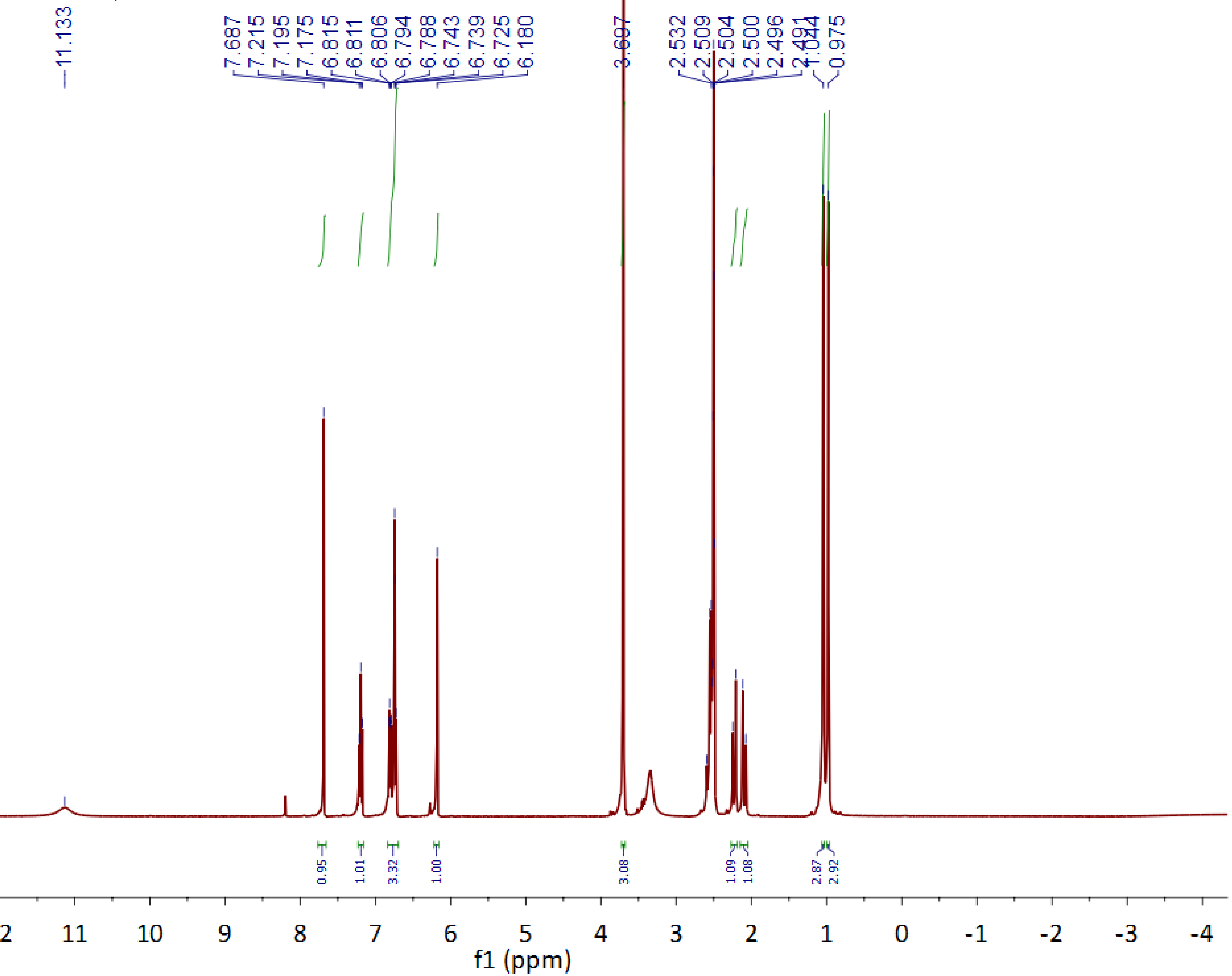

^{13}C NMR (100 MHz, DMSO-d$_6$)

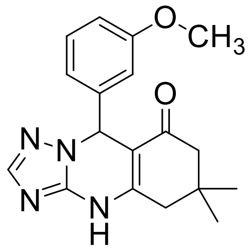

4g

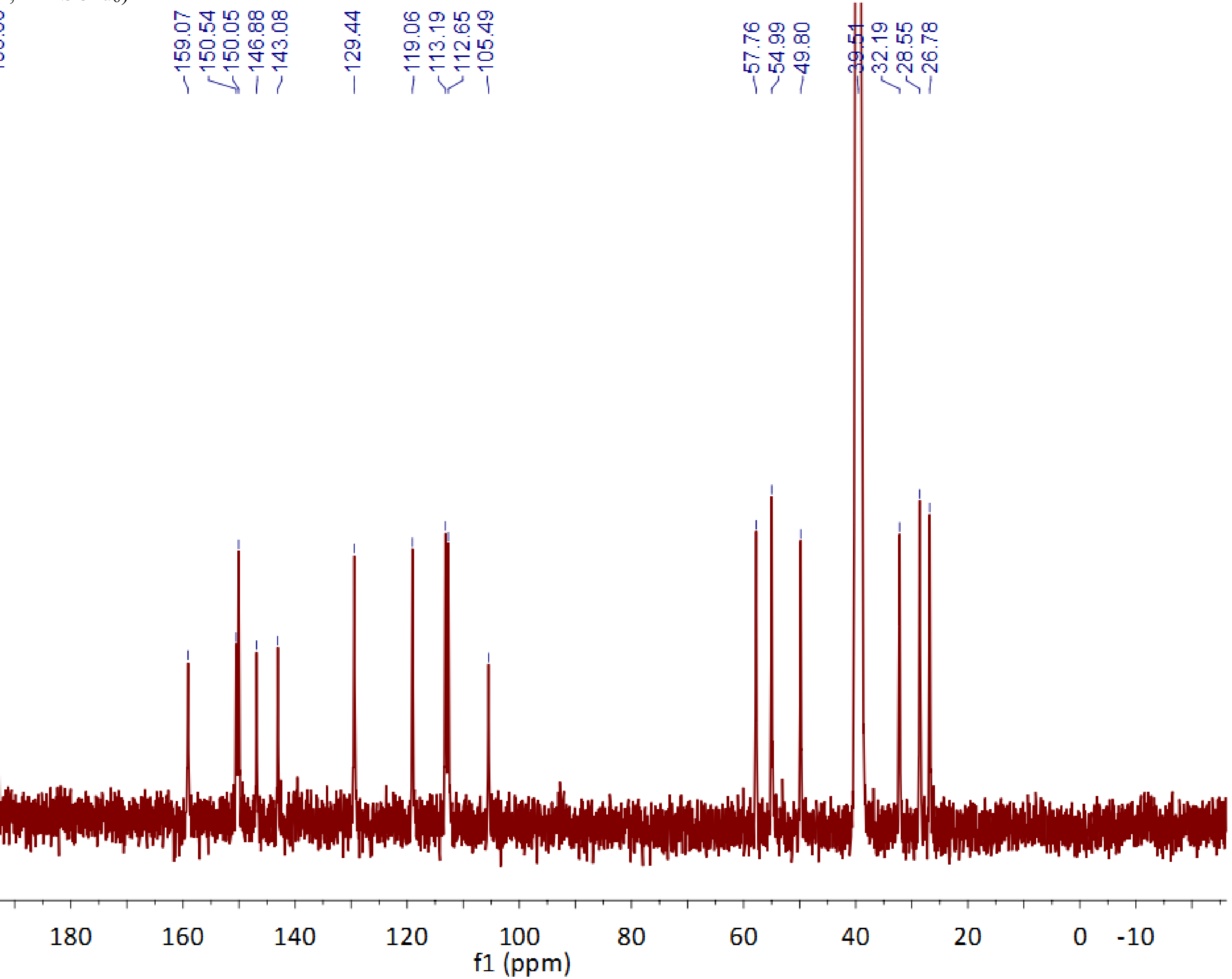

^{1}H NMR (400 MHz, DMSO-d$_6$)

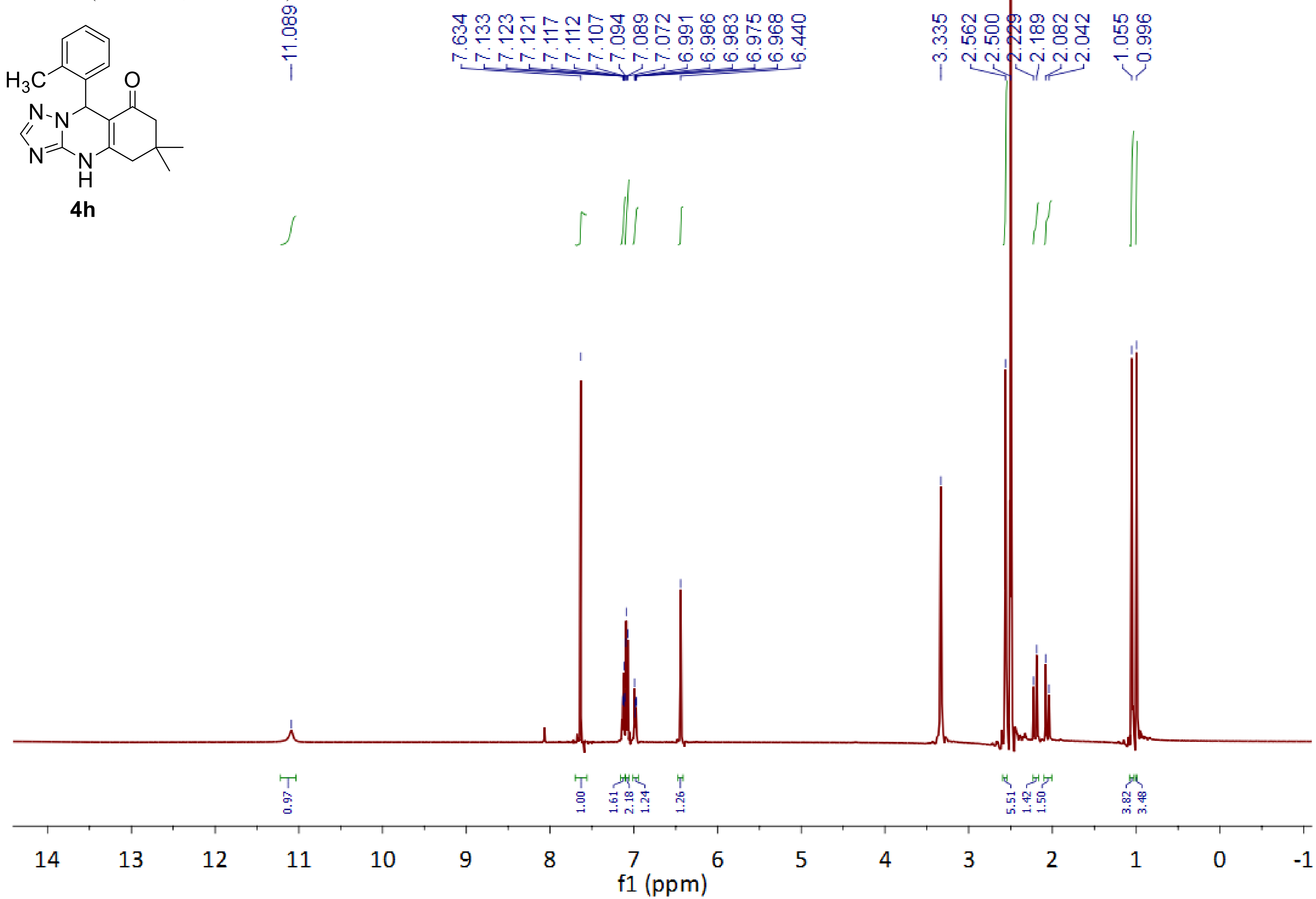

^{13}C NMR (100 MHz, DMSO-d$_6$)

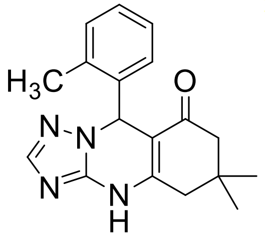

4h

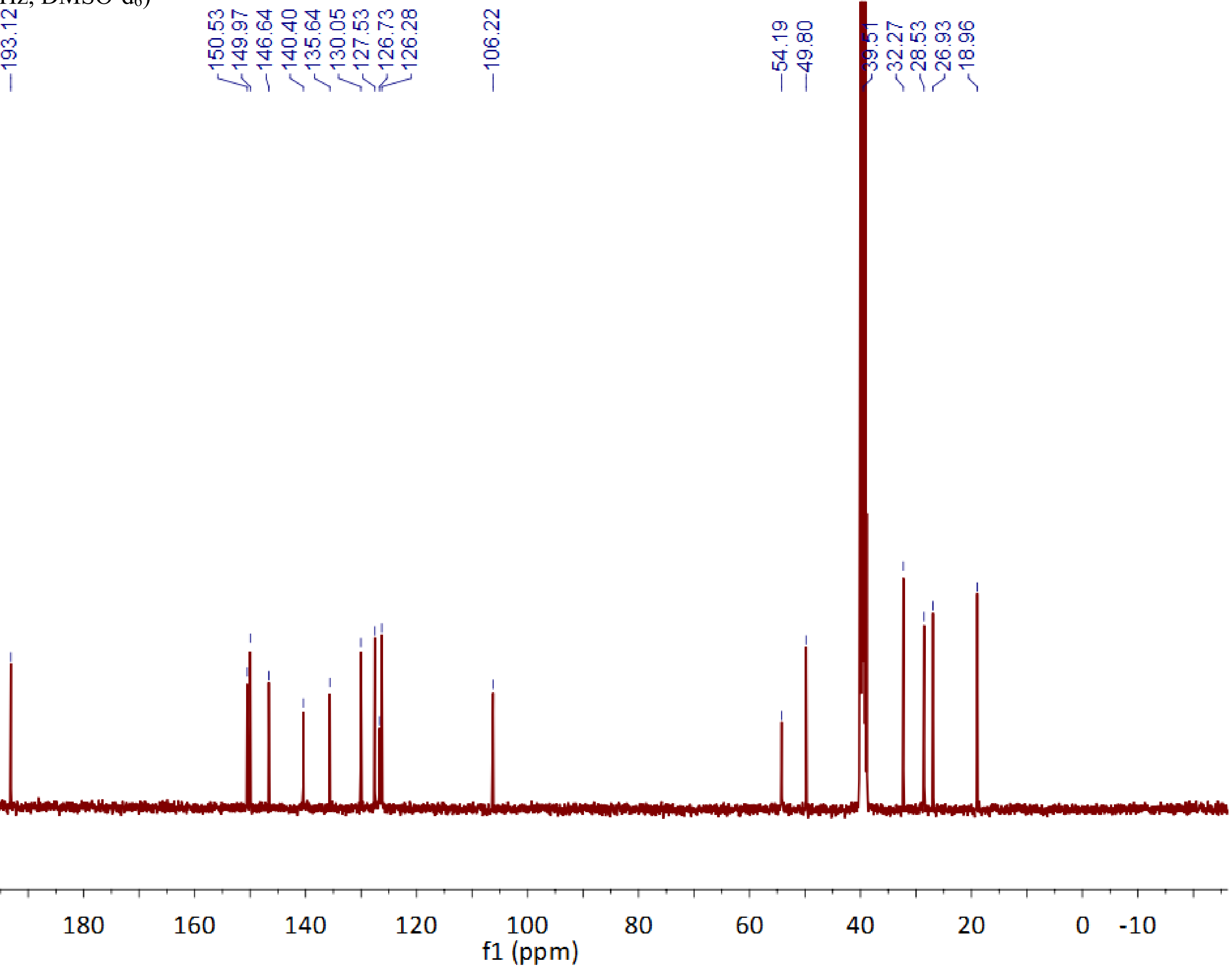

^{1}H NMR
(300 MHz, DMSO-d_6)

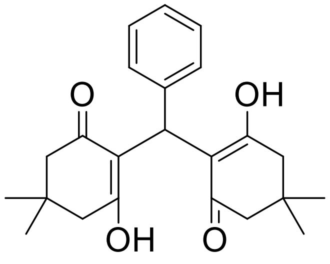

5'

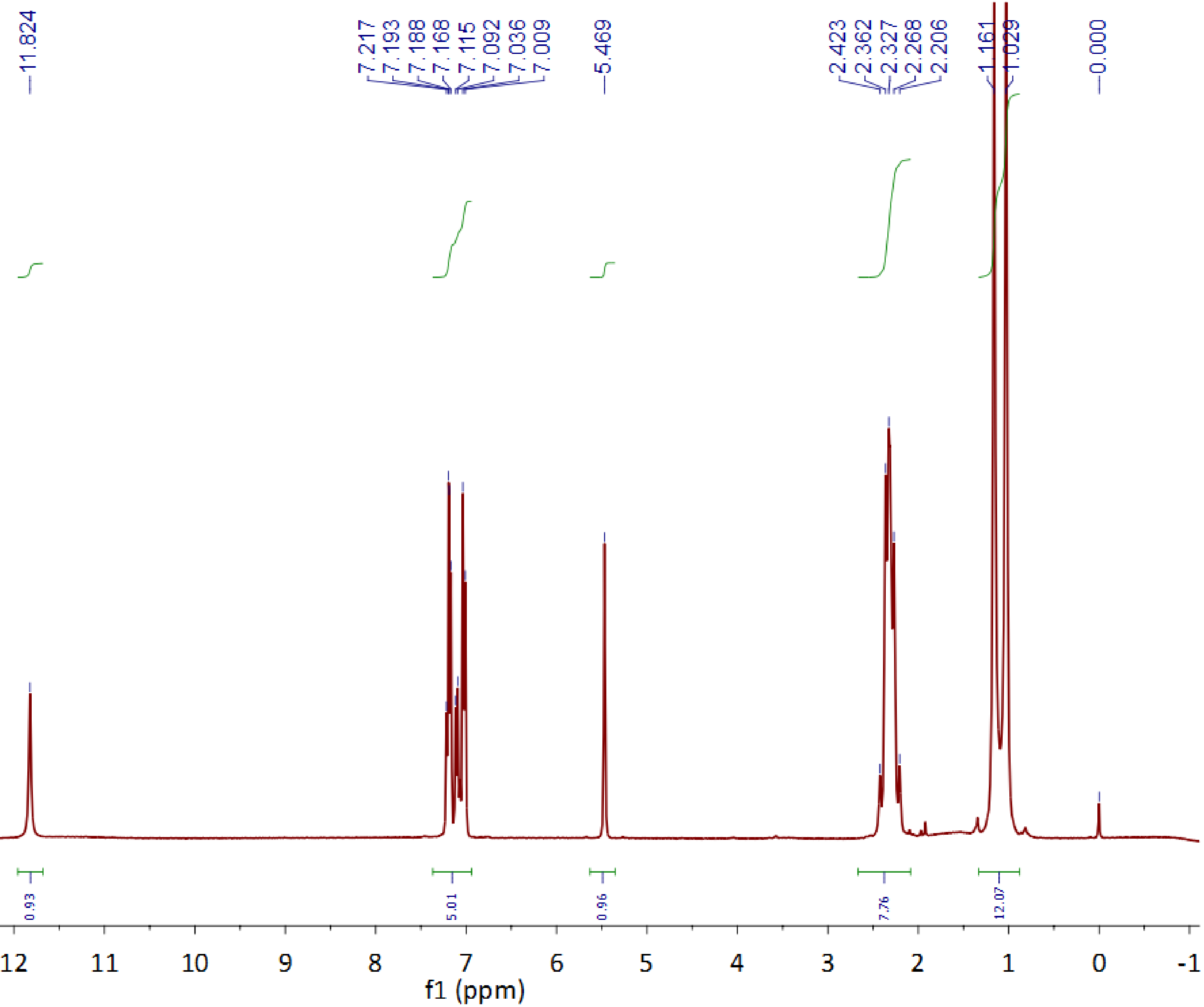

^{13}C NMR
(75 MHz, DMSO-d_6

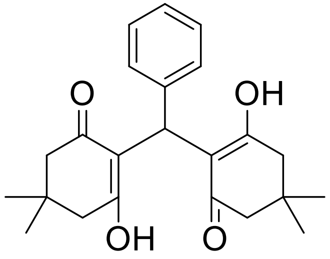

5'

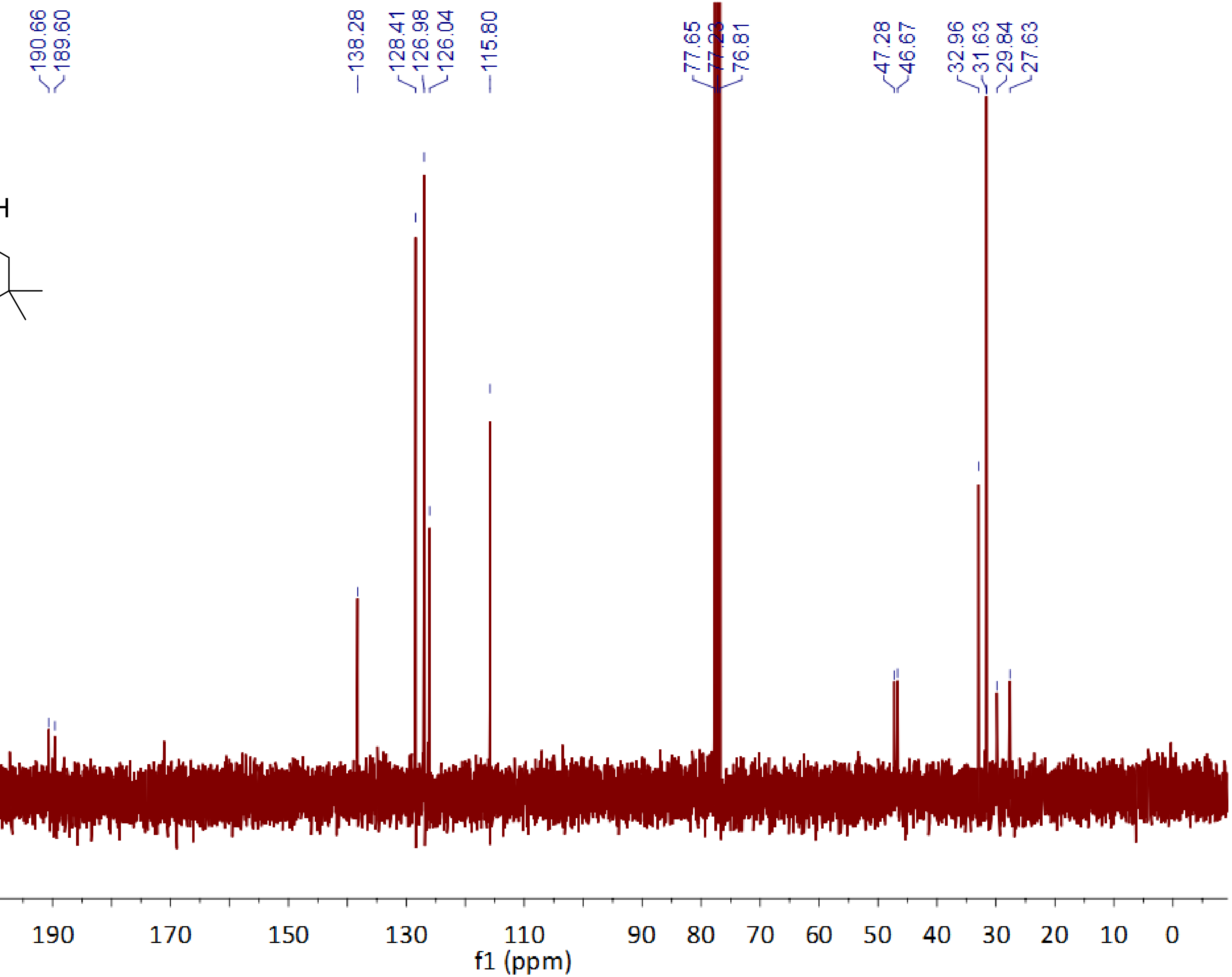

Single Crystal XRD data:

CCDC 1897344 contains the supplementary crystallographic data for **4c**. This data can be obtained free of charge from The Cambridge Crystallographic Data Centre via www.ccdc.cam.ac.uk/structures

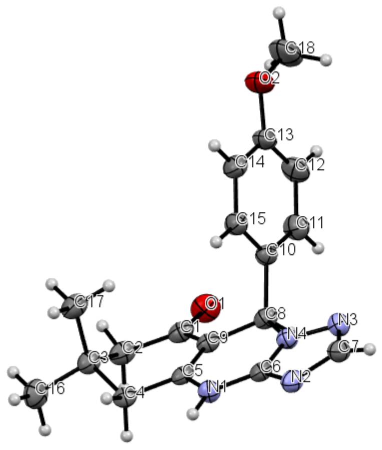

Figure S4. ORTEP drawing and labeling scheme of **4c**.

Table S3. Crystal data and experimental details for **4c**.

Empirical formula	C18 H20 N4 O2	
Formula weight	324.38	
Crystal system	Monoclinic	
Space group	C2/c	
Unit cell dimensions	a = 23.7257(13) Å	= 90°.
	b = 5.9658(3) Å	= 104.386(6)°.
	c = 26.8496(17) Å	= 90°.
Volume	3681.2(4) Å^3	
Z	8	
F(000)	1376	
Density (calculated)	1.171 Mg/m^3	
Wavelength	0.71073 Å	
Cell parameters reflections used	1517	
Theta range for Cell parameters	3.5470 to 25.9790°.	
Absorption coefficient	0.079 mm^{-1}	
Temperature	200(2) K	
Crystal size	0.25 x 0.20 x 0.15 mm^3	
Data collection		
Diffractometer	Xcalibur, Atlas, Gemini	
Absorption correction	Semi-empirical from equivalents	
Max. and min. transmission	1.00000 and 0.91296	
No. of measured reflections	6679	
No. of independent reflections	3223 [R(int) = 0.0470]	
No. of observed [I>2_igma(I)]	1963	
Completeness to theta = 24.997°	99.4 %	
Theta range for data collection	3.194 to 24.997°.	
Refinement		
Final R indices [I>2sigma(I)]	R1 = 0.0498, wR2 = 0.0982	
R indices (all data)	R1 = 0.0963, wR2 = 0.1201	
Goodness-of-fit on F^2	0.974	
No. of reflections	3223	
No. of parameters	217	
No. of restraints	0	
Largest diff. peak and hole	0.186 and -0.213 e.Å^{-3}	

Preliminary results for the synthesis of 6,6-dimethyl-9-(pyridin-3-yl)-5,6,7,9-tetrahydro-[1,2,4]triazolo[5,1-*b*]quinazolin-8(4*H*)-one.

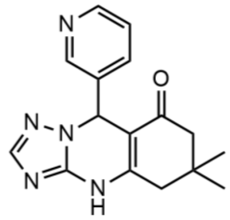

Our preliminary tests show that the reaction procedure also works with heterocyclic aldehydes such as pyridine-3-carbaldehyde yielding the corresponding triazoloquinazolinone. Synthesis of 6,6-dimethyl-9-(pyridin-3-yl)-5,6,7,9-tetrahydro-[1,2,4]triazolo[5,1-*b*]quinazolin-8(4*H*)-one was carried out using the general procedure. Product was purified by recrystallization in ethanol yielding white solids (181.4 mg, 61% yield). ^{1}H and ^{13}C NMR data are shown below.

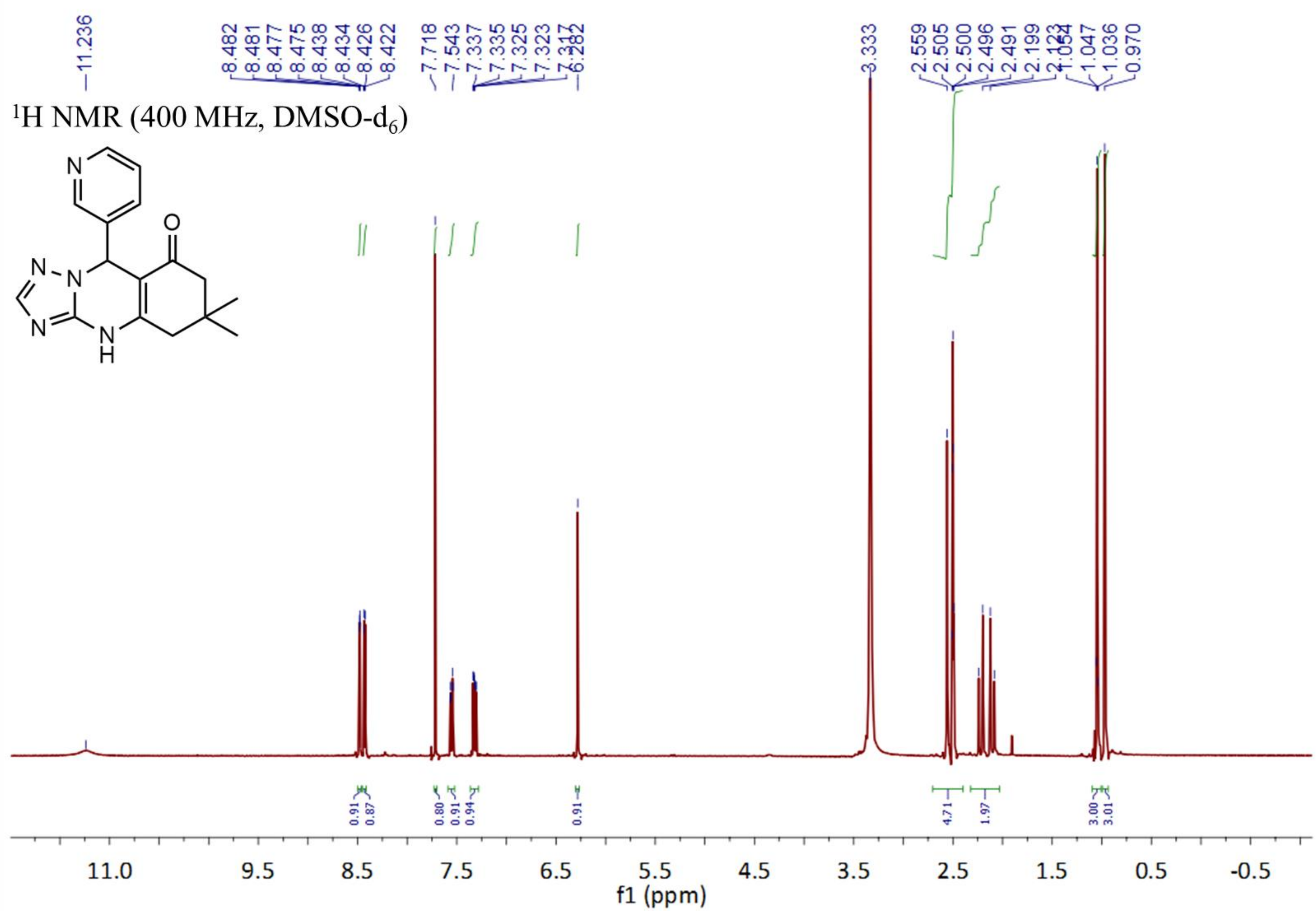

^{1}H NMR (400 MHz, DMSO-d$_6$)
11.236
8.482
8.481
8.477
8.475
8.438
8.434
8.426
8.422
7.718
7.543
7.337
7.335
7.325
7.323
7.317
6.282
3.333
2.559
2.505
2.500
2.496
2.491
2.199
2.123
1.054
1.047
1.036
0.970
0.91
0.87
0.80
0.91
0.94
0.91
4.71
1.97
3.00
3.01
11.0
9.5
8.5
7.5
6.5
5.5
4.5
3.5
2.5
1.5
0.5
-0.5
f1 (ppm)
N
O
N
N
N
N
H

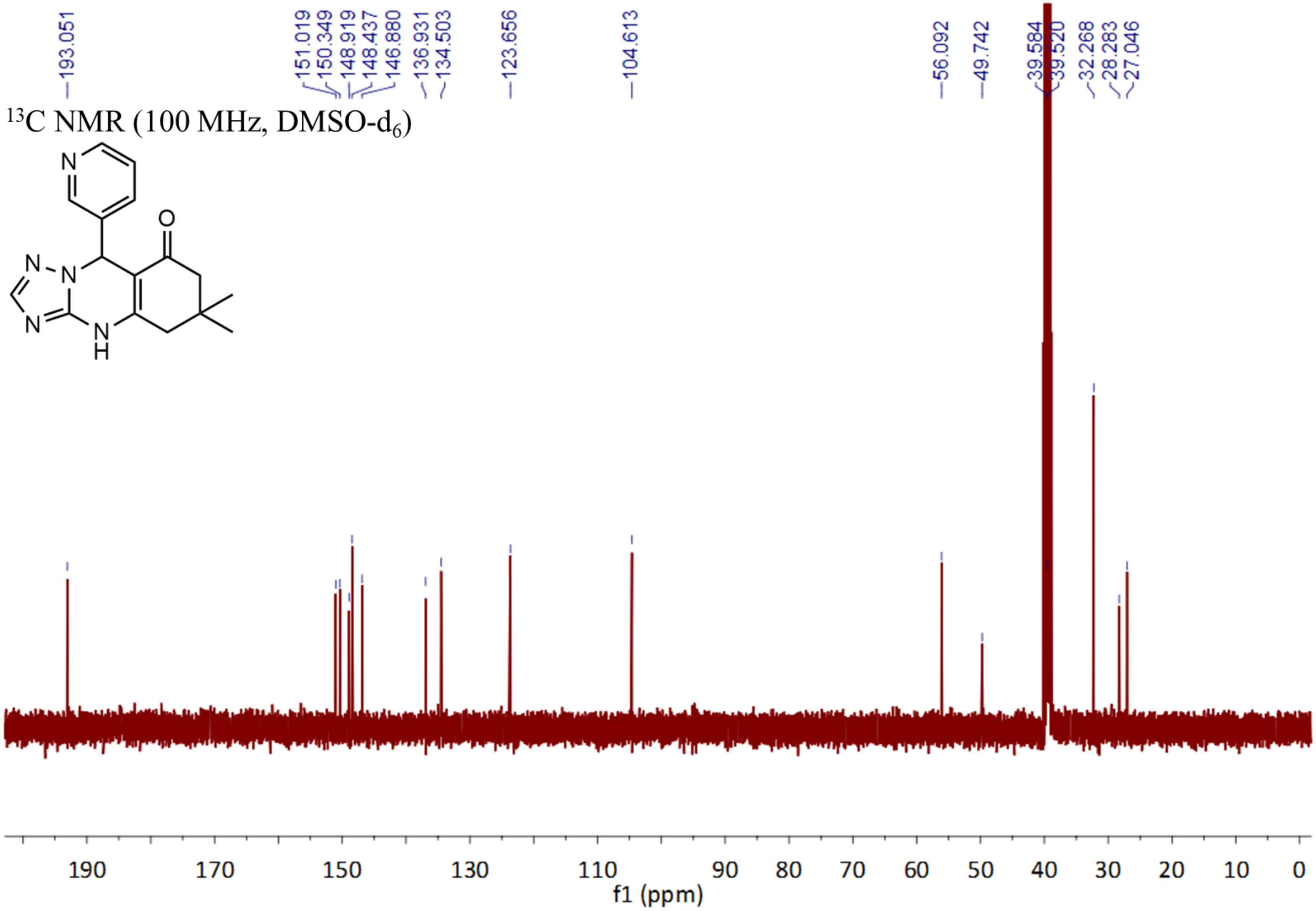

193.051
151.019
150.349
148.919
148.437
146.880
136.931
134.503
123.656
104.613
56.092
49.742
39.584
39.520
32.268
28.283
27.046
^{13}C NMR (100 MHz, DMSO-d_6)
N
O
N
N
N
N
H
190
170
150
130
110
90
80
70
60
50
40
30
20
10
0
f1 (ppm)

References

1 Chen, C.-H. *et al.* Effective Synthesis of Highly Oxidized Graphene Oxide That Enables Wafer-scale Nanopatterning: Preformed Acidic Oxidizing Medium Approach. *Sci. Rep.* **7**, 3908 (2017).

2 Hummers, W. S. & Offeman, R. E. Preparation of Graphitic Oxide. *J. Am. Chem. Soc.* **80**, 1339-1339 (1958).

3 Gao, W., Alemany, L. B., Ci, L. & Ajayan, P. M. New insights into the structure and reduction of graphite oxide. *Nature Chemistry* **1**, 403 (2009).

4 Heravi, M. M., Derikvand, F. & Ranjbar, L. Sulfamic Acid-catalyzed, Three-Component, One-pot Synthesis of 1,2,4 Triazolo/Benzimidazolo Quinazolinone Derivatives. *Synth. Commun.* **40**, 677-685 (2010).

5 Ahmad, S., Elham, F. & Abbas, R. Synthesis of Tetrahydrobenzimidazo[1,2-b]quinazolin-1(2*H*)-one and Tetrahydro-1,2,4-triazolo[5,1-b]quinazolin-8(4*H*)-one Ring Systems Under Solvent-Free Conditions. *Comb. Chem. High Throughput Screen.* **9**, 771-776 (2006).

6 Sharghi, H., Aboonajmi, J., Aberi, M. & Shiri, P. Heterogeneous $AlPO_4(SO_3H)$ nanosheets: novel catalyst for the multi-component synthesis of quinazolinones and highly functionalized piperidines. *J. Iran. Chem. Soc.* **15**, 1107-1118 (2018).

7 Yu, J.-J. *et al.* Synthesis of tetraketones in water and under catalyst-free conditions. *Green Chem.* **12**, 216-219 (2010).
